# Alginate-Based Ternary Composites for Water Treatment: Synthesis, Mechanisms, and Applications

**DOI:** 10.3390/polym18161941

**Published:** 2026-08-07

**Authors:** Jia Li, Alzhan Baimenov, Jechan Lee, Seitkhan Azat

**Affiliations:** 1 Laboratory of Engineering Profile, Satbayev University, Almaty 050013, Kazakhstan; em7839821-d@stud.satbayev.university (J.L.); alzhan.baimenov@satbayev.university (A.B.); 2 School of Energy and Constructional Engineering, Shandong Huayu University of Technology, Dezhou 253034, China; 3 Department of Global Smart City, School of Civil, Architectural Engineering, and Landscape Architecture, Sungkyunkwan University, Suwon 16419, Republic of Korea

**Keywords:** binary composites, ternary composites, engineering application, water treatment

## Abstract

Alginate, as a natural polymer, has been widely used in the removal of pollutants in water due to its renewability, biocompatibility and abundant functional groups. However, it still has some limitations such as low specific surface area, poor mechanical strength and single function. Introducing metal oxides can effectively enhance their adsorption capacity and multi-functionality. Nevertheless, binary composites remain insufficient for treating complex water bodies. Therefore, the construction of structurally stable and functionally diverse ternary composites has become an important research direction. The review is based on alginate/metal oxide binary composites, analyzing their deficiencies in structural stability, nanoparticle dispersion and functional synergy. On this basis, the synthetic strategy and structural characteristics for constructing ternary composites by incorporating inorganic non-metallic frameworks, metal nanoparticles, porous carbon-based materials, and other natural polymers are discussed. The synergistic mechanism and performance enhancements of various components during pollutant removal are primarily summarized. Although the ternary composite system has a promising application prospect, it still faces challenges such as complex synthesis processes, insufficient interfacial stability, limited reusability, and poor adaptability to complex water bodies. Therefore, future efforts should focus on structural optimization, green preparation and resource utilization of waste materials to promote their engineering application and sustainable development in the field of water treatment.

## 1. Introduction

### 1.1. Water Pollution and Sustainable Water Treatment Technologies

With the continuous acceleration of industrialization, urbanization and agricultural intensification, a large amount of industrial wastewater, domestic sewage and agricultural chemicals is continuously discharged into aquatic environments, leading to increasingly serious water pollution problems [1]. In recent years, the types of pollutants detected in water environments have been increasing continuously, including organic dyes, antibiotics, pesticides, personal care products, polycyclic aromatic hydrocarbons and other organic pollutants [2], as well as heavy metals [3] and microplastic pollutants [4]. Given the characteristics of these pollutants, such as persistence, bioaccumulation, potential toxicity and poor biodegradability, once released into aquatic systems, they are prone to accumulate along the food chain, posing significant risks to ecosystem stability and sustainable human development [5]. Therefore, it is crucial to develop sustainable alternative solutions and efficient methods for removing such pollutants, including the use of environmentally friendly processes, the utilization of low-cost or recycled waste [6], and the avoidance of secondary pollution [7].

Currently, common water treatment methods for water pollution problems include adsorption, membrane separation, coagulation–sedimentation, chemical oxidation, electrochemical methods, and biological treatment methods [8]. However, with the diversification and complexity of water pollution, traditional treatment methods have gradually exposed some limitations. Among various water treatment technologies, adsorption has become one of the most promising technologies in the field of water pollution control due to simple operation, high efficiency, wide applicability and relatively low cost. In recent years, researchers have tended to use biological adsorbents that are harmless to the environment and do not require chemical reagents, such as nutshells, food waste, agricultural waste, as well as natural polymer adsorbents, including sodium alginate, chitosan, and clay [9]. Natural biopolymers (cellulose, alginate, chitosan, etc.) [10] have been proven to be highly efficient adsorbents for removing pollutants from water due to their excellent physical and chemical stability, biodegradability [11], low cost, non-toxicity, and environmental sustainability [12].

Cellulose has mechanical strength, but contains relatively few active functional groups and usually requires further chemical modification. Chitosan is rich in amino groups and has a strong complexing ability for heavy metal ions, but its stability is limited under acidic conditions. Starch and carboxymethyl cellulose are low-cost and have good biocompatibility, but their poor water resistance and limited mechanical strength restrict their practical applications. In contrast, sodium alginate is not only rich in carboxyl and hydroxyl groups, but also can rapidly undergo ionic crosslinking with divalent metal ions under mild conditions, forming a stable three-dimensional hydrogel structure.

**Figure 1:**
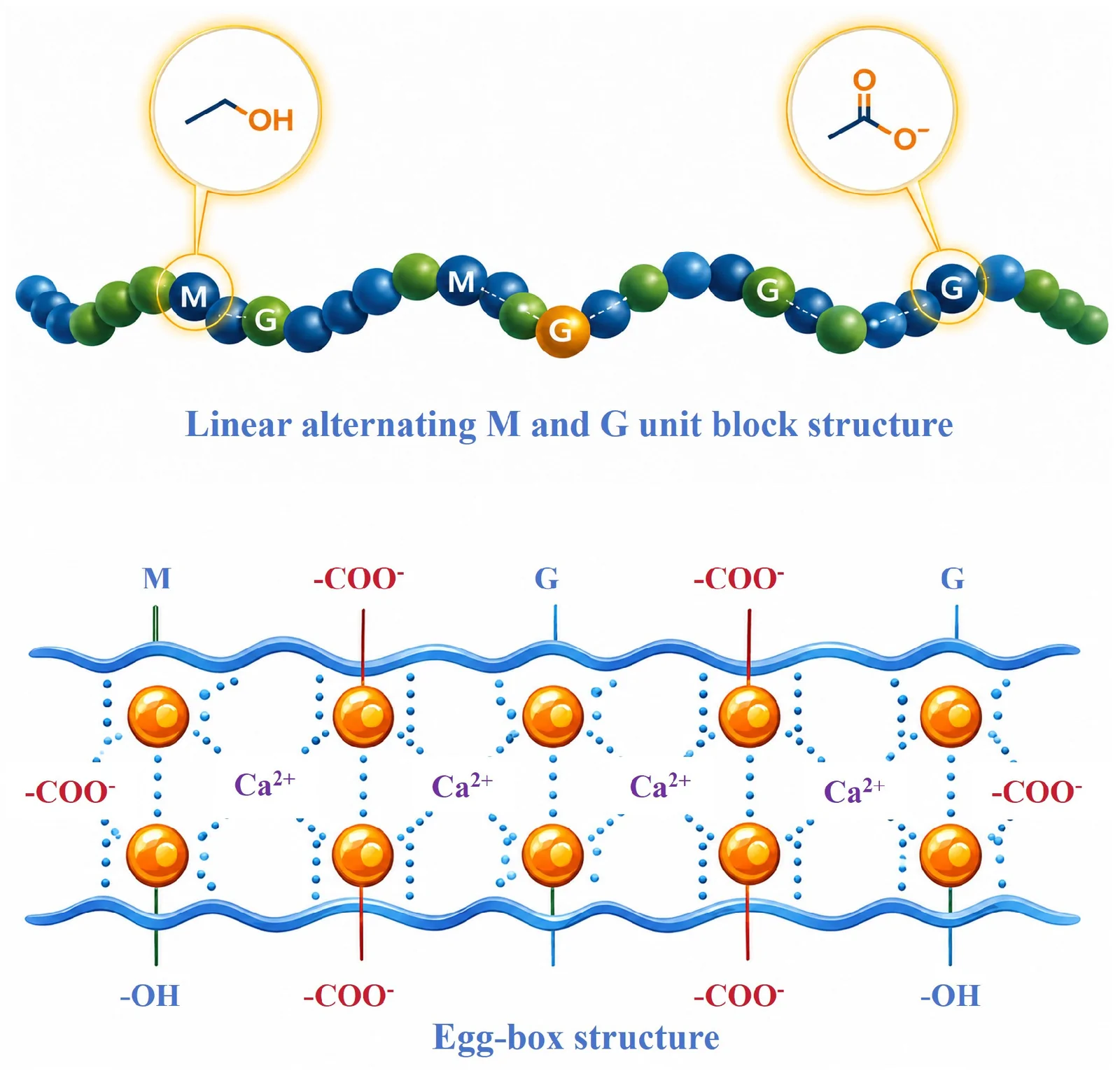
Egg-box structure of sodium alginate.

Sodium alginate (SA), derived from algae, is a naturally abundant and renewable natural polymer polysaccharide [13]. The SA molecular structure is rich in manuronic acid (M) and gururonic acid (G) units. The Na^+^ on G units can undergo ion exchange reactions in the presence of divalent or higher-valent metal cations (except Mg^2+^ and Hg^2+^), and further crosslink to form a stable gel structure [14]. In this process, G units in alginate chains are bridged by multivalent metal ions, forming a three-dimensional network hydrogel structure [15]. Within the alginate gel structure, the –COOH on adjacent SA chains are connected through hydrogen bonding, while the polymer chains are crosslinked with multivalent bonds formed by M+. This interchain structure has large gaps and is usually referred to as the “egg-box structure” (Figure 1). Benefiting from its unique three-dimensional network structure, sodium alginate has hydrophilicity, gel-forming ability, biocompatibility, non-toxicity and biodegradability. In addition, SA has abundant oxygen-containing functional groups such as carboxyl groups (–COOH) and hydroxyl groups (–OH), enabling efficient adsorption of diverse pollutants through electrostatic interaction, complexation, and hydrogen bonding [16]. Therefore, sodium alginate-based materials have received increasing attention in water treatment.

### 1.2. Sodium Alginate as a Bio-Based Adsorbent

In the field of water treatment, SA is usually prepared into diverse physical forms such as microspheres, hydrogel membranes, hollow fibers, and nanofibers to meet different application requirements [17]. Although SA has rich –COOH and –OH functional groups and outstanding gel-forming performance, there are still certain limitations in practical applications, including a limited diversity of functional groups, restricted specific surface area and pore structure [18], insufficient mechanical stability [19], and limited functionality, which limit their practical application in complex environments [20].

Based on the three-dimensional network structure and abundant oxygen-containing functional groups of SA, composite modification can be achieved by introducing graphene, biochar, natural minerals, metal oxides, organics, and functional microorganisms [21], and this composite modification strategy integrates the advantages of different materials, thereby overcoming the limitations of SA in terms of structural stability, specific surface area, functionality, and reusability through synergistic effects [22].

### 1.3. SA/Metal Oxide Binary Composites

Among the numerous materials compounded with SA, nano-metal oxides have been widely used in the functional modification of SA due to their unique surface properties, abundant active sites and high specific surface area. Metal oxides can not only significantly enhance the adsorption performance and mechanical stability of SA, but also endow the composites with multiple functionalities, including photocatalysis [23], antibacterial activity [24], magnetic separation [25] and catalytic reduction [26]. Meanwhile, the three-dimensional network structure of SA can serve as a support for metal oxide nanoparticles, enhancing their structural stability and reusability [27], thereby further improving the overall performance of the composites.

This synergy effect has demonstrated outstanding performance in water treatment applications. Wang et al. [28] prepared core–shell SA@AM composite nanospheres by dispersing amino-modified magnetic nanoparticles (AMs) in a sodium alginate solution, followed by Ca^2+^-induced ionic crosslinking in a CaCl_2_ solution. The results showed that the maximum adsorption capacity of SA@AM for Pb^2+^ reaches 105.8 mg/g, which is 50.1% higher than pure SA. Even after adsorption–desorption cycles, the removal rate remains above 76%, effectively overcoming the shortcomings of traditional adsorbents, such as difficult recovery and low reusability. Abbas et al. [29] synthesized biogenic ZnO nanoparticles using Bacillus cereus and immobilized them in calcium alginate beads to prepare recyclable adsorption–photocatalytic materials for treating dye wastewater. Ai et al. [30] further coated activated Al_2_O_3_ in CA to construct an Al_2_O_3_/CA composite adsorbent. Compared with Al_2_O_3_, the Al_2_O_3_/CA composite has a larger specific surface area (increasing from 37.31 m^2^/g to 51.73 m^2^/g), and can achieve a 100% removal rate within 12 h at an initial phosphate concentration of 2.5 mg/L. Moreover, it shows stable performance in fixed-bed dynamic adsorption and enabling efficient removal of phosphorus from water bodies.

**Figure 2:**
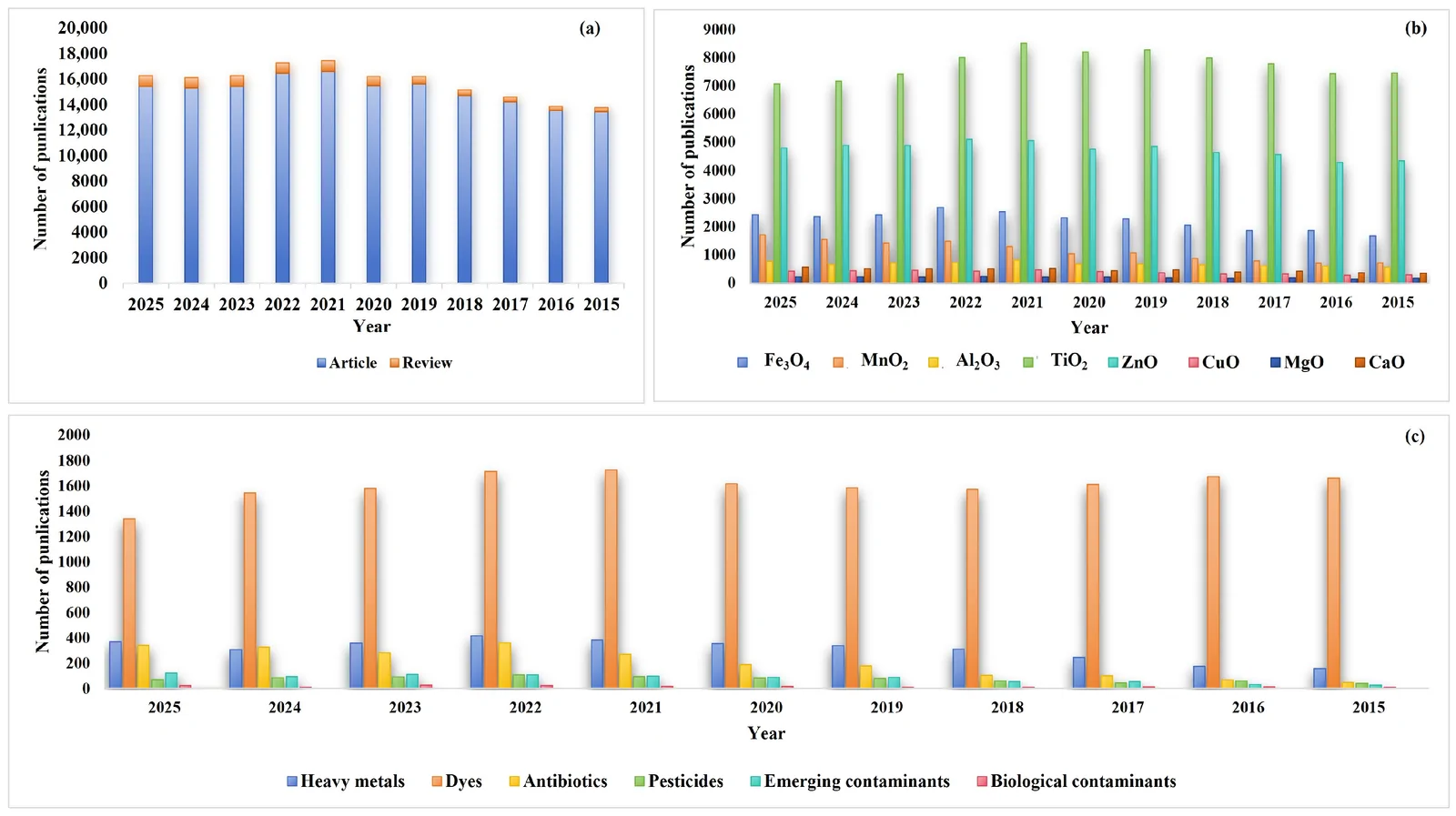
The trends in research on alginate-based metal oxide composites based on the Web of Science Core Collection database: (**a**) the annual publication output from 2015 to 2025; (**b**) the distribution of studies involving different metal oxides; and (**c**) the proportion of research on composites applied to the treatment of different types of water pollutants.

Currently, research on SA/metal oxide composites has become a significant direction in the field of water treatment. Based on the Web of Science Core Collection database, we used “alginate” and “metal oxide” as the core keywords, combined with relevant screening conditions, to conduct a statistical analysis of publications from 2015 to 2025. As shown in Figure 2a, the number of related article publications increased steadily from 2015 to 2021, reaching a peak around 2021, followed by a slight decline and stabilization, indicating that this field has gradually moved from a rapid development stage to a relatively mature stage, and continues to attract significant research attention. Further analysis reveals (Figure 2b) that the research hotspots mainly focus on adsorption performance optimization, magnetic separation, photocatalytic degradation, and the construction of multi-functional composites. Among them, metal oxides such as TiO_2_, ZnO, Fe_3_O_4_, MnO_2_, and Al_2_O_3_ have been widely introduced into alginate-based systems due to their efficient adsorption, magnetic, photocatalytic, and antibacterial properties, as well as their adjustable structure, which achieves a synergistic enhancement of material performance. Overall, the field is evolving from single adsorption functionality to multi-functional integration, showing a clear trend of cross-integration. From an application perspective (Figure 2c), adsorption remains the primary mechanism for pollutant removal, while photocatalysis and antibacterial functions serve as important supplementary approaches, promoting the development of materials towards multi-functional synergy.

Several reviews [1,11,12,20,21,22] have summarized the preparation and environmental applications of alginate-based adsorbents, hydrogels, and nanocomposites. However, these reviews mainly focus on general alginate-based materials, binary composite systems, or individual pollutant removal processes. A systematic review specifically addressing alginate-based ternary composites remains lacking, particularly regarding construction strategies, the roles of third components, synergistic mechanisms, quantitative binary–ternary comparisons, regeneration, and practical applicability. Therefore, this review aims to fill this gap by providing a systematic and critical evaluation of alginate-based ternary composites for water treatment.

### 1.4. Motivation for Developing Ternary SA-Based Composites

Although the combination of metal oxides into SA can enhance material performance and multi-functional synergistic effects, there is still room for further improvement. The main limitations can be summarized as follows: (i) Metal oxide nanoparticles still have agglomeration phenomena in the composite system, resulting in a decrease in the utilization of active sites [31]. (ii) The stability and dispersibility of metal oxides still require further improvement [32]. (iii) The SA structure has insufficient stability and is prone to swelling or damage [33]. (iv) The SA adsorption site type is single and has poor adaptability to complex pollutants. (v) The specific surface area and pore structure of the composite are limited, leading to unsatisfactory mass transfer performance [34]. (vi) The regenerative ability and cycle stability are limited [35]. Therefore, the development of multi-component composite systems with enhanced structure stability and richer functions has become a research hotspot.

Among various modification strategies, composite modification based on the introduction of functional components is an important approach. Common modified materials mainly include inorganic non-metallic frameworks [36], metal nanoparticles, porous carbon-based materials [37], and natural polymers. Firstly, metal nanoparticles can not only endow the composite system with catalytic activity, antibacterial properties or magnetic functionality, but also improve the overall performance of the material through electron transfer or synergistic effects.

Secondly, inorganic non-metallic framework materials (e.g., SiO_2_, zeolites, and bentonite) can serve as structural control units, providing a stable supporting framework for the composite system. Among them, SiO_2_ is particularly widely used in multi-component systems due to its outstanding chemical stability, adjustable pore structure, and abundant surface Si-OH groups [38]. SiO_2_ can inhibit agglomeration and improve the dispersion of metal oxides [39], strengthen the three-dimensional network structure, and enhance porosity and mass transfer efficiency. Cha et al. [40] prepared Fe_3_O_4_@SiO_2_ nanoparticles with a core–shell structure using sodium silicate as the silicon source, demonstrating that the SiO_2_ shell improved the dispersion stability, chemical stability, and magnetic properties of Fe_3_O_4_@SiO_2_ core–shell nanoparticles. Similarly, Han et al. [41] used rice husk ash as an SiO_2_ source and natural SA as a reinforcing component to prepare SA–SiO_2_ (SAS) composite aerogels through a sol–gel and freeze-drying method for efficient adsorption of organic pollutants. In the composite, SiO_2_ serves as the matrix skeleton, providing a high specific surface area and a porous structure. SA significantly enhances the mechanical strength and structural stability of the aerogel, while also regulating the pore volume and pore size distribution.

In addition, porous carbon-based materials (such as activated carbon and biochar) with high specific surface area and abundant pore structure can provide additional adsorption sites and mass transfer channels for the system, not only improving the dispersion stability of the composite but also enhancing the adsorption capability in synergy with the sodium alginate matrix [42]. Li et al. [43] used waste reed biochar as the matrix and played a crucial role in Fe/(MgFe_2_O_4_-BC) composite microspheres. The results indicate that biochar can act as both a support and a pore-forming agent in the microsphere structure, further promoting the dispersion of nanoparticles during the pyrolysis process.

Other natural polymers can also be introduced [44]. Such materials can be constructed by constructing crosslinked networks or interpenetrating polymer networks (IPNs) [45], achieving precise regulation of the architecture [46]. Among them, cellulose nanocrystals or chitosan molecular chains can form stable networks with sodium alginate and pre-complex metal ions by abundant functional groups (such as –OH, –COOH, and –NH_2_), thereby regulating the particle size distribution and spatial dispersion of metal oxides during in situ growth [47]. Overall, different types of components play a synergistic role in structural regulation, performance enhancement and functional expansion, promoting the development of alginate-based composites towards high efficiency and multi-functionality.

The review is based on binary composite systems of SA and metal oxides, analyzes their limitations in structural stability and dispersion, and further explores the modification strategies for constructing ternary composite systems by introducing inorganic non-metallic frameworks, metal nanoparticles, porous carbon-based materials, and natural polymers. The paper introduces the synthesis methods and structural characterization of different composites, and systematically expounds the synergistic mechanism and performance advantages of different components, including adsorption capacity, mechanical strength, reusability, and environmental safety. The practical application potential of alginate-based composite adsorbents in the water treatment fields is comprehensively evaluated, such as heavy metals, dyes, and antibiotics, providing valuable insights for the design and application of high-performance ternary alginate-based composites.

## 2. Synthesis of Alginate-Based Composites

### 2.1. Synthesis of SA/Metal Oxide Binary Composites

During the construction of SA/metal oxide binary composites, different synthesis methods have a significant impact on the structural characteristics, component distribution and interfacial interactions of the materials. Currently, the common synthetic methods include ionic crosslinking and in situ synthesis [48]. These two methods have significant differences in terms of operational complexity, particle dispersion and interfacial bonding strength, which in turn have different effects on the performance of composites. Therefore, conducting a systematic analysis of different synthetic methods is of great significance for understanding the structure–performance relationship of materials and optimizing material design.

#### 2.1.1. Ionic Crosslinking Method

**Figure 3:**
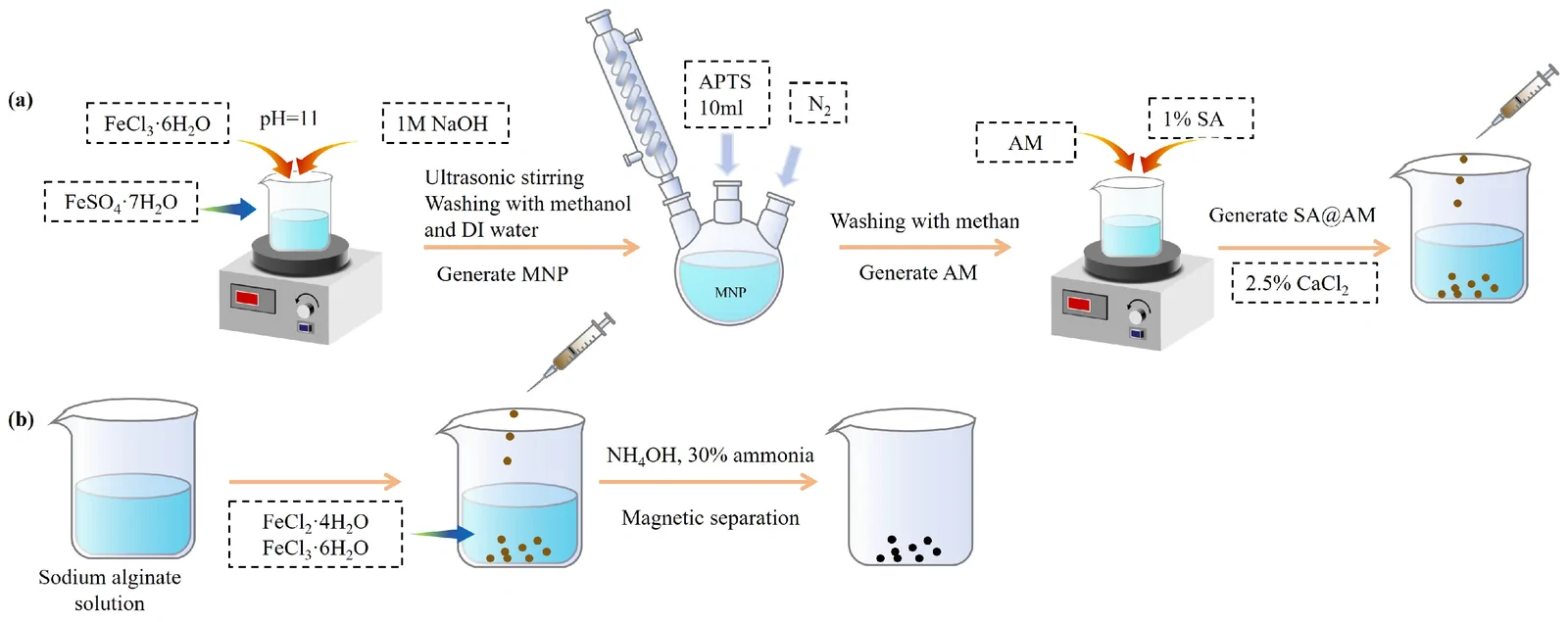
(**a**) Fabrication of magnetic polyelectrolyte microspheres (SA@AM); (**b**) in situ preparation of Fe_3_O_4_–alginate hydrogel magnetic beads.

The ionic crosslinking method is a commonly used method for constructing SA/metal oxide composites [49]. The fundamental principle involves dispersing the pre-synthesized metal oxide nanoparticles in SA solution and forming a three-dimensional gel network through ionic crosslinking, thereby achieving the fixation of functional components, and solving problems such as metal leaching and separation difficulties [50]. Wang et al. prepared Fe_3_O_4_ magnetic nanoparticles (MNPs) by the co-precipitation method [28], and obtained positively charged AM with APTES. Subsequently, these particles combined with SA (1%, *w*/*v*) through electrostatic interactions, followed by ionic crosslinking in CaCl_2_ solution (2.5%, *w*/*v*), to form a new type of magnetic SA polyelectrolyte composite nanosphere (SA@AM). The synthesis graphics are shown in Figure 3a. The FTIR results indicated that SA@AM exhibits characteristic absorption peaks corresponding to Fe–O, N–H and Si–O–C. The enhanced absorption peak at 3397 cm^−1^ in SA@AM was consistent with the characteristics of SA, indicating that Fe_3_O_4_ was successfully aminated and further combined with SA. The successful construction of the composite has been confirmed. The XRD results show that the crystalline structure of magnetic Fe_3_O_4_ remains unchanged during the surface modification process, which is beneficial for maintaining its paramagnetic properties. BET analysis showed that the specific surface area gradually increased from MNP (58.72 m^2^/g) to AM (72.69 m^2^/g) and SA@AM (104.85 m^2^/g), indicating that surface modification and ion crosslinking significantly improved structural properties and provided more active sites for ionic crosslinking. The VSM analysis indicated that SA@AM still maintained paramagnetization, but the magnetization intensity decreased (6.85 emu/g), indicating that SA successfully coated the particle surface. The Zeta potential further confirms the formation of the composite structure, as the surface charge shifts from AM (+8.41 mV) to SA@AM (−15.09 mV). The TEM results show that MNPs are spherical with uniform size, but the particles are prone to agglomeration. The surface of AM becomes dense and amorphous. SA@AM presents regular nanospheres (15–22 nm). The TEM images of SA@AM show a high electron density, and the edges are semi-transparent spheres, presenting a typical three-dimensional network and core–shell structure. The structure effectively enhances the structural stability, specific surface area and mechanical strength of the material, thereby achieving efficient adsorption performance.

Safav et al. [51] synthesized Fe-Mn bimetallic oxides by the REDOX method and prepared iron–manganese–alginate macroporous microspheres by ionic crosslinking with CaCl_2_ (0.1 M) and CA (2.5%, *w*/*v*). FTIR detected characteristic functional groups such as –OH, –COO^−^, Fe–O, and Mn–O, confirming the successful loading of metal oxides. SEM revealed a rough surface morphology with a macroporous structure, with irregular cavities and connected channels, which is conducive to the diffusion of arsenic ions and the utilization of internal active sites. The BET results showed a relatively low specific surface area (0.0836 m^2^/g), but with an average pore size of approximately 34 nm, presenting a typical macroporous structure. This is mainly attributed to the dense alginate matrix, which restricts mesoporous development but forms a more open mass transfer network, effectively reducing diffusion resistance. Meanwhile, the alginate matrix can inhibit the agglomeration of nanoparticles and enhance the accessibility of active sites. In terms of performance, Fe provides the main adsorption sites, while Mn enhances oxidation and synergistic adsorption capacity, resulting in significantly improved performance compared with single-metal systems. This material reaches adsorption equilibrium within 150 min, exhibits rapid kinetic behavior, and has a maximum adsorption capacity as high as 71.4 mg/g. Overall, this material achieves an efficient, rapid and environmentally friendly arsenic removal process through the bimetallic synergy and an alginate-based support matrix. However, its specific surface area is relatively low, and the lack of application verification for regeneration indicates that further optimization is still required.

Monroy et al. [52] constructed SA/TiO_2_ hydrogel microspheres with adsorption–photocatalytic functions by introducing TiO_2_ into sodium alginate using CaCl_2_ ionic crosslinking. During preparation, the suspension is treated with refrigeration to remove air bubbles, thereby improving the uniform dispersion of TiO_2_ within the microspheres and enhancing their mechanical stability. The degradation experiment shows that when the TiO_2_ loading is increased from 10% to 30%, the degradation rate of ciprofloxacin rises from 87.5% to 100%. However, when the loading reaches 30%, TiO_2_ leaching was observed in the system, indicating that the excessive catalyst will cause the coordination sites in SA to tend to saturation, and some unbound TiO_2_ will be lost during the reaction process. Therefore, considering the overall performance, a 20% load is regarded as the optimal condition. XRD analysis indicates that the crystalline structure of TiO_2_ remained unchanged before and after the introduction into the SA matrix, indicating good photocatalytic stability. The SEM results show that the microspheres remain spherical both before and after use, with a diameter of 2–3 mm. However, after recycling, obvious pores appeared in the hydrogel matrix. And magnified images revealed aggregation of TiO_2_ particles on both the surface and inside the structure. Although SA/TiO_2_ composites exhibit significant advantages in terms of structural stability and functional synergy, there are still issues of particle agglomeration and leaching under high-load conditions, indicating that the binary system still needs further optimization in terms of interface bonding strength and structural regulation. Similarly, Wan et al. [53] prepared TiO_2_/SA microspheres by using the ionic crosslinking with divalent metal ions (e.g., Cu^2+^, Co^2+^, and Sr^2+^), achieving the immobilization and recyclability of powder photocatalysts. The performance results show that the degradation rate of methyl orange by Sr-TiO_2_ hydrogel microspheres under UV irradiation is as high as 99% for 60 min. The degradation rate is superior to that of Cu-TiO_2_ (120 min) and Co-TiO_2_ (130 min), and can be comparable to the activity of pure TiO_2_ powder. Moreover, after 10 cycles, the degradation rate remains above 95% with excellent stability.

For the ionic crosslinking method, the selection of ionic crosslinking agents is very important. Many divalent and trivalent metal ions can be used as ionic crosslinking agents for sodium alginate, and their main characteristics are summarized as shown in Table 1. Overall, Ca^2+^ is currently the most widely used crosslinking agent due to its low cost, good biocompatibility and mild crosslinking conditions. Ba^2+^ has a higher binding affinity with G units, resulting in a more stable gel network. However, due to its high toxicity, its application is limited. In addition to providing ionic crosslinking effects, Zn^2+^ can also endow materials with antibacterial activity and adsorption performance. In comparison, trivalent metal ions such as Fe^3+^ and Al^3+^ can generate a higher crosslinking density, enhancing the mechanical stability of the materials. However, excessive crosslinking may lead to an increase in gel brittleness and limit the diffusion of contaminants within the material. In addition, the concentration of the crosslinking agent and the crosslinking time also significantly affect the gel structure. When the concentration is too low or the crosslinking time is insufficient, the network structure is difficult to adequately develop. However, excessively high crosslinker concentrations or prolonged crosslinking times may result in the formation of an overly dense outer layer, reducing porosity and mass transfer efficiency. Therefore, the type, concentration and crosslinking time of crosslinking agents need to be reasonably optimized based on the material composition and target application.

In conclusion, the ionic crosslinking method can effectively immobilize and disperse metal oxide nanoparticles into an alginate system, endowing the composites with good structural stability, recyclability and adsorption–catalytic–antibacterial synergistic functions [54]. However, existing research has found that this method is prone to particle agglomeration and metal leaching under high-loading conditions. Meanwhile, the restriction of pore structure by the alginate matrix leads to a relatively low specific surface area, which in turn affects mass transfer and the utilization of active sites. Furthermore, due to the relatively weak interfacial binding, the material is prone to particle loss during multiple recycling processes, thereby limiting its cycling stability and causing a significant decline in treatment efficiency. Therefore, the binary composite system constructed by the ionic crosslinking method still has considerable room for optimization in terms of structural regulation and mass transfer performance.

#### 2.1.2. In Situ Synthesis Method

To overcome limitations such as poor dispersion and insufficient interfacial binding in the ionic crosslinking method, researchers have proposed the in situ method. The method involves introducing metal ion precursors into a sodium alginate solution, and through chemical reactions (adjusting pH or adding precipitants), metal oxides are in situ generated in the sodium alginate matrix, thereby achieving a more uniform distribution. Chen et al. [55] prepared CaO_2_/SA microspheres by in situ synthesis using SA as the matrix and employed CaCl_2_, H_2_O_2_, and NaOH as precursors. During the preparation process, the solutions containing SA and NaOH were gradually added drop by drop to a solution of CaCl_2_ and H_2_O_2_. Under strongly alkaline conditions, CaO_2_ particles were generated in situ and uniformly coated. After curing for 4 h, structurally stable microspheres were formed. Through the tests and comparisons of H_2_O_2_ release capacity and phosphorus removal capacity, the optimal conditions were SA (2%, *w*/*v*), NaOH (0.2 M), CaCl_2_ (0.1 M), and H_2_O_2_ (0.1 M). A low H_2_O_2_ concentration resulted in insufficient CaO_2_ loading, while a high concentration led to structural damage of the microsphere. The moisture content of the microspheres is approximately 99%. The high moisture content is conducive to the slow release of H_2_O_2_ and biocompatibility. The material exhibited remarkable phosphorus removal performance within a wide pH range of 3.0 to 10.0, with a maximum removal efficiency of up to 97%, and can continuously release H_2_O_2_ (0.06 mM, up to 26 d). Meanwhile, it achieves efficient inhibition of Microcystis aeruginosa, nearly complete removal within 24 h, and the algae inhibition effect can last for 170 days, demonstrating superior synergistic algae and phosphorus removal capabilities. XRD and SEM analyses confirmed that the CaO_2_ crystal phase was successfully generated in the microspheres, with a layered three-dimensional structure on the surface and uniform particle distribution. Combining XRD and EDS analyses indicated that the particles were mainly composed of CaO_2_ and contained a small amount of CaOH. Compared with the ionic crosslinking method, this method enables a more uniform distribution of CaO_2_ in the microspheres without aggregation, leading to improved utilization of active sites. However, the in situ synthesis method is highly dependent on reaction conditions, and the controllability and universality of the system are still limited. Moreover, it mostly focuses on the construction of binary structures, and there are deficiencies in the coordinated regulation of multiple components.

Soumia et al. [56] prepared Fe_3_O_4_/SA nanocomposite magnetic microspheres (Fe_3_O_4_ NPs-ALG) by an in situ synthesis method (Figure 3b). Firstly, a mixed iron salt solution containing FeCl_3_ and FeCl_2_ was prepared as a crosslinking agent for SA and a precursor for Fe_3_O_4_. Then, 2% (*w*/*w*) SA solution was added dropwise to the mixed iron salt solution (room temperature, pH = 6.1) to form hydrogel microspheres. Subsequently, ammonia water (NH_4_OH, 30%) was added in situ to generate Fe_3_O_4_ nanoparticles. XRD results show that the characteristic diffraction peak of Fe_3_O_4_ was consistent with the standard card, indicating that Fe_3_O_4_ has been successfully generated. The particle size of pure Fe_3_O_4_ nanoparticles is approximately 20 nm, while that of the composite was about 9 nm, indicating that the alginate network effectively restricts crystal growth. FTIR analysis indicated that the composite retains the characteristic absorption peaks of alginate (O–H, CH_2_, COO^−^ and C–O), and a Fe-O vibration peak appeared at 566 cm^−1^, further confirming the formation of Fe_3_O_4_. SEM results show that the surface of the microspheres is rough and wrinkled, and a typical nanofibrous network structure is formed inside. EDS analysis detected O and Fe elements and indicated a relative excess of oxygen, suggesting that Fe_3_O_4_ is coordinated with the alginate matrix through coordination. The absorption peaks at 310 nm and 450 nm in the ultraviolet–visible diffuse reflection spectrum further confirm the existence of Fe_3_O_4_.

Aiming to address the problems of traditional alginate extraction using organic solvents, including the low mechanical strength of pure alginate, difficult separation and high cost, Lucaci et al. [57] prepared an Alg–Fe_3_O_4_ biosorbent via in situ synthesis directly in alginate extract derived from algae. In total, 3.976 g of FeCl_2_ and 10.812 g of FeCl_3_, respectively, were dissolved in 100 mL alginate extract. The two solutions were mixed in a volume ratio of 2:1, the pH was adjusted to 11 with 1 M NaOH, the mixture was stirred vigorously for 1 h, and the obtained microspheres were vacuum filtered. Characterization through FTIR, SEM-EDS and BET confirmed that the alginate was successfully loaded on the surface of the Fe_3_O_4_ microspheres. The material presented a spherical porous structure with a specific surface area of 136.18 m^2^/g. The alginate fixation rate exceeded 92%, and good stability in water. Batch adsorption experiments were carried out with Cu (II), Co (II), and Zn (II) as target pollutants. When the metal ion concentration was lower than 0.80 mmol/L, the removal rates reached 99%, 89%, and 95%, respectively, and the removal rate was still higher than 75% after five cycles. Overall, the results demonstrate that the material is environmentally friendly in preparation, has outstanding adsorption capability and reusability, and holds great potential for application in the treatment of heavy metal wastewater.

The in situ synthesis method achieves the in situ growth and uniform dispersion of active components in a three-dimensional network by generating metal oxides in situ in the alginate matrix, effectively avoiding the common issues of particle agglomeration and weak interfacial binding force in ionic crosslinking methods. This method not only significantly enhances the structural stability and interfacial interaction of the composite but also regulates the size of nanoparticles, thereby improving the utilization rate of active sites and functional synergy. However, compared with the ionic crosslinking method, the in situ synthesis method is more dependent on reaction conditions (such as pH, precursor concentration and reaction pathway), making the system regulation more complex. Challenges remain in terms of reproducibility and large-scale preparation. In addition, its applicability is relatively limited, with current studies mainly focusing on binary composites, and further development is still needed for multi-component synergistic construction and functional expansion.

In addition to the ionic crosslinking method and the in situ synthesis method, in recent years, new composite methods such as the emulsion crosslinking method, microfluidic techniques and spray-drying method have also been developed [58], which have great advantages in structure regulation, particle size control and interface bonding. Penas-Nunez et al. [59] prepared TiO_2_/SA microspheres using a microfluidic technique. A mixed SA/TiO_2_ solution was used as the dispersed phase, and uniform droplets were formed by gas flow shearing in the microfluidic chip and then dropped into the CaCl_2_ solution for crosslinking to form microspheres. This method can achieve precise control of particle size ranging from tens to hundreds of μm, which is conducive to increasing the specific surface area and exposing more active sites, thereby overcoming the problem of uneven particle size in traditional dropwise methods. In addition to the microfluidic technique, emulsion crosslinking and spray-drying methods also offer good particle size control capabilities and structural designability in the construction of alginate-based materials. However, these methods often involve organic solvents or complex equipment and may face particle agglomeration or interfacial compatibility issues during the introduction of metal oxides. Consequently, their application in this system remains relatively limited and requires further exploration.

The construction of alginate-based composites has formed multiple preparation paths represented by ionic crosslinking, in situ synthesis, and emerging structure-regulation methods. The ionic crosslinking is simple to operate under mild conditions and is suitable for the rapid compounding of different nanomaterials. However, it generally has limitations such as poor particle dispersion, weak interfacial binding, low specific surface area, metal oxide leaching, and insufficient cycling stability [60]. The in situ synthesis method effectively improves the dispersion and interfacial interaction of nanoparticles by generating metal oxides within the SA matrix. However, this method is sensitive and dependent on reaction conditions, and precise control over structural uniformity remains challenging. In addition, emerging methods represented by microfluidic techniques have demonstrated obvious advantages in particle size regulation and structural uniformity and provided a new technical route for the refined construction of SA composites.

Considerable progress has been achieved in the structural construction of SA/metal oxide binary composite systems. On the one hand, alginate provides abundant –COOH, –OH, and a three-dimensional network structure, serving as a remarkable adsorption matrix and support to improve its structural stability and reusability. On the other hand, the introduction of metal oxides endows the material with magnetism, photocatalytic activity, antibacterial properties or selective adsorption capacity for specific pollutants, thereby achieving synergistic performance enhancement. However, the binary system with SA/metal oxide still has certain limitations. The binary functional components have difficulty in meeting the multiple demands for adsorption, catalysis and stability in complex aqueous environments. Moreover, there is still considerable room for optimization in aspects such as increasing specific surface area, regulating pore structure, and the efficient utilization of functional sites. In addition, issues such as nanoparticle aggregation, insufficient utilization of active sites, and declining long-term cycling performance are still observed in some systems. Therefore, based on the research foundation of binary systems, introducing a third component to construct ternary composites with multi-level structures and multi-functional synergistic effects helps to regulate the pore structure and specific surface area of the materials, enhance structural stability, solve the problems of metal oxide agglomeration and leaching, and achieve multi-mechanism synergistic effects such as adsorption, catalysis, magnetic separation, and antibacterial activity, to provide a theoretical basis for the rational design of high-performance materials for efficient water treatment.

### 2.2. Synthesis of Ternary Composites

The preparation of ternary composites is typically based on binary composites. By introducing a third component, such as inorganic non-metallic frameworks, metal nanoparticles, porous carbon-based materials, and natural polymers, multi-level structural optimization and functional synergistic enhancement can be achieved. From the perspective of synthesis routes and process complexity, the synthesis methods of ternary composites can be classified into two categories: The first is the one-step composite approach; through the blending–ionic crosslinking method and the blending self-assembly method, the synchronous introduction and structural construction of multiple components are achieved in the one-step process. The second is the stepwise composite process, which usually takes a pre-constructed binary system as the basis and introduces a third component in combination with in situ loading or functionalization modification steps.

Due to the differences in structural characteristics and functional mechanisms of various third components, the corresponding preparation methods also differ. Therefore, there are significant differences in regulating the structural hierarchy, interfacial interactions, and the distribution of functional components. The following sections will take material types as the main line and further conduct a systematic review in each component material category in combination with specific synthesis methods, to reveal the relationship between different preparation methods and the regulation of material structure and performance.

#### 2.2.1. Ternary Systems Based on Inorganic Non-Metallic Frameworks

In ternary composite systems, inorganic non-metallic frameworks are widely used to optimize the structure and performance of SA/metal oxide binary systems due to their high structural stability, large specific surface area and adjustable pore structure. Among them, SiO_2_, zeolite and bentonite are the three most representative materials, but their construction approaches and functional mechanisms exhibit notable differences.

In recent years, SiO_2_ has been widely utilized in the construction of multi-functional composites due to its efficient chemical stability, adjustable pore structure and abundant Si–OH. SiO_2_ can provide a large specific surface area, abundant pore structure and surface active sites, thereby contributing to the enhancement of structural stability and adsorption performance of the composites. Notably, in addition to traditional chemical methods, biomass resources such as agricultural waste can also be used as silicon sources to prepare bio-based SiO_2_, providing a new approach for the green preparation and high-value utilization of SiO_2_ [61]. However, at present, most composite systems still mainly rely on chemical methods to synthesize SiO_2_.

**Figure 4:**
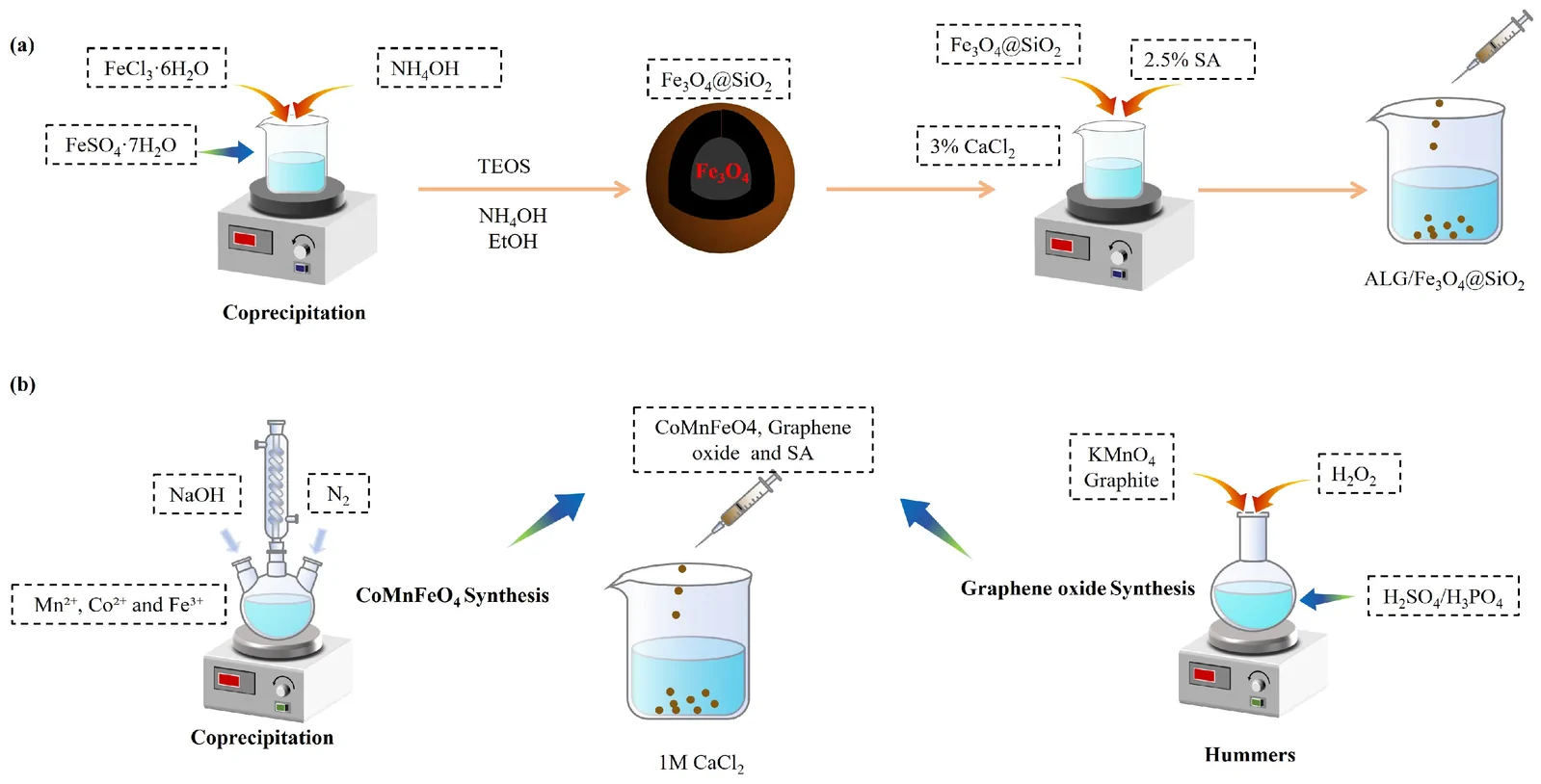
(**a**) The preparation of ALG/Fe_3_O_4_@SiO_2_ by the ion crosslinking method; and (**b**) a schematic representation of the synthesis procedure for Alg-GO-CoMnFeO_4_ beads.

SiO_2_ is usually introduced into composite systems as a structural support and dispersion carrier. Composed of a Si-O-Si skeleton and rich in -Si-OH on its surface, SiO_2_ can combine with alginate molecular chains through hydrogen bonding or electrostatic interactions, thereby enhancing interfacial interactions. In terms of synthesis methods, ionic crosslinking or blending self-assembly methods are often adopted. Firstly, metal oxide/SiO_2_ nanoparticles are generated and then uniformly dispersed in alginate solution to construct a structurally stable and well dispersed ternary composite system. Barzegarzadeh et al. [62] synthesized Fe_3_O_4_ by a co-precipitate method, and then obtained Fe_3_O_4_@SiO_2_ core–shell nanoparticles by the sol–gel method with SiO_2_ coating. Subsequently, SA (2.5%, *w*/*v*) was mixed with Fe_3_O_4_@SiO_2_ (0.5%, *w*/*v*) and then dropped into a 3% CaCl_2_ solution (100 mL) for crosslinking to form microspheres. After water washing and drying at 35 °C, composite microspheres were obtained (Figure 4a). XRD analysis indicated that the ALG/Fe_3_O_4_@SiO_2_ was successfully formed, and the SiO_2_ coating did not alter the crystal structure of Fe_3_O_4_. FESEM shows that the Fe_3_O_4_ particles are uniform size of 70–90 nm, and after modification with SiO_2_, the sizes increased to 100–180 nm. The Fe_3_O_4_@SiO_2_ particles are uniformly dispersed in a spherical form within the alginate matrix. EDS results further confirmed the presence of C, O, Si, Cl, Na, Fe, and Ca elements in the microspheres, verifying the successful introduction of Fe_3_O_4_@SiO_2_, sodium alginate and crosslinking agent. VSM analysis shows that the saturation magnetization of Fe_3_O_4_@SiO_2_ and ALG/Fe_3_O_4_@SiO_2_ is 10.85 emu/g and 2.68 emu/g, respectively. The decrease in saturation magnetization is attributed to the introduction of SA components, but ALG/Fe_3_O_4_@SiO_2_ still has sufficient magnetism. The rapid separation of microspheres can be achieved through an external magnetic field. In terms of adsorption capability, the maximum adsorption capacity for chlorpyrifos is as high as 154.2 mg/g, significantly superior to multiple adsorbents reported in the existing literature. After eight adsorption–desorption cycles, it still retains an adsorption efficiency of 74.9%, demonstrating good reusability. The ternary composite has advantages such as rapid magnetic separation, high adsorption capacity, efficient stability and reusability. In this system, SiO_2_ plays a crucial role by inhibiting the agglomeration of Fe_3_O_4_ nanoparticles; maintaining the magnetic response characteristics; significantly enhancing the mechanical strength, chemical stability, and structural durability of microspheres; and increasing the number of adsorption sites and strengthening interfacial interactions, thereby synergistically improving both adsorption capacity and reusability.

MCM-41, with a well-ordered mesoporous structure, high specific surface area and abundant Si-OH groups, can play multiple roles such as structural support, dispersion, fixation, and stability enhancement in ternary composite systems. Hachemaoui et al. [63] used Fe_3_O_4_, MCM-41 and SA as raw materials. Fe_3_O_4_ was first synthesized by the precipitation method, then mechanically mixed to obtain Fe_3_O_4_@MCM-41(x) composites (x = 0.25, 0.5, 1 g), and subsequently mixed with sodium alginate solution (2%, *w*/*v*). Hydrogel microspheres were prepared via ionic crosslinking using a 4% (*w*/*v*) CaCl_2_ solution, and finally obtained MC@CA (0.25), MC@CA (0.5), and MC@CA (1) aerogel microspheres through freeze-drying. The SEM results showed that the introduction of Fe_3_O_4_@MCM-41 significantly increased the surface roughness of the microspheres, with MC@CA (1) exhibiting the most pronounced effect. The magnetic composites were uniformly attached to the pore walls, and as their content increased, the pore size of the microspheres decreased, and the surface coverage increased. FTIR analysis indicated that the ternary composites simultaneously contained characteristic absorption peaks such as –OH, –COO^−^, T–O–T (Si–O–Si/Al–O–Si) and Fe–O, indicating that all components were successfully integrated into the system. Some characteristic peaks in the composites showed intensity changes and overlapping, especially the –COO^−^ absorption peak shifted, indicating the interaction between the -COOH in alginate and the surface -OH of the inorganic components. The above results demonstrate that the ternary system not only effectively integrated all components but also formed stable interfacial interactions, which were conducive to the construction and performance improvement of the composite structure. XPS results further confirmed the presence of elements such as Si, Al, Fe, C, O, Ca, and Na, indicating that Fe_3_O_4_@MCM-41 was successfully coated by CA. In the UV–Vis spectra, the broad absorption band was attributed to peak overlap arising from interactions among multiple components, consistent with the FTIR and XPS results. In this study, MCM-41 effectively solved the easy aggregation of Fe_3_O_4_ nanoparticles through its high specific surface area and ordered pore structure, achieving uniform distribution of active sites. At the same time, MCM-41 as an “intermediate layer” anchored the magnetic components in the calcium alginate matrix, not only enhancing the porosity of the composites to facilitate the diffusion of reactants, but also significantly suppressing iron ion leaching, thereby maintaining high catalytic efficiency while improving the structural stability and environmental safety.

Liu et al. [64] constructed a ternary composite system using a green blending self-assembly method, with Fe_3_O_4_, SiO_2_ and SA as raw materials, without an external linking agent. Firstly, Fe_3_O_4_@SiO_2_ core–shell particles were prepared by the sol–gel method, and then directly mixed and stirred with sodium alginate aqueous solution (3%, *w*/*v*) to form Fe_3_O_4_@SiO_2_@ SA (GMS) magnetic nano-adsorbents. TEM and SEM results show that GMS has a uniform spherical structure with a particle size of approximately 100 nm, and is well dispersed without obvious agglomeration. FTIR analysis indicated that the characteristic absorption peaks of –OH, –COO^−^, Fe–O, and Si–O–Si were simultaneously present in the composites, confirming the successful composite of each component. Notably, the absorption peak of –COO^−^ shifts to 1624 cm^−1^, indicating a hydrogen bond interaction between the carboxyl group of alginate and the silanyl group on the surface of Fe_3_O_4_@SiO_2_. Furthermore, the appearance of Si–O–C and Si–O–Si/C–O overlapping peaks further indicates that SA has successfully bound to the surface of Fe_3_O_4_@SiO_2_. VSM analysis shows that the magnetic intensifications of Fe_3_O_4_, Fe_3_O_4_@SiO_2_, and GMS are 83.5, 79.9, and 75.0 emu/g, respectively. The materials possess remarkable magnetic responsiveness and can achieve rapid magnetic separation. Performance evaluation shows that after being recycled eight times, the removal rate still remains above 80%, demonstrating superior stability and reusability.

Zeolite is a typical type of crystalline aluminosilicate porous material, with a Si–Al–Si tetrahedral skeleton structure. featuring high specific surface area, regular and orderly pore structure, remarkable ion exchange performance and good mechanical stability. In a ternary composite system, zeolite not only serves as a stable and rigid framework to enhance the structural strength of the alginate matrix, but also can achieve the screening and enrichment of target pollutants through its regular pores. Meanwhile, the abundant surface active sites of zeolite are conducive to forming a synergistic effect with metal oxides, which can not only improve the dispersion and stability of metal oxides, but also further enhance the adsorption capacity and selectivity of the material. Ternary systems containing zeolites can usually be efficiently constructed through a simple ionic crosslinking method, which has the advantages of a simple process and strong operability.

Gao et al. [65] used ZrO_2_ and zeolite molecular sieve (ZMS) as functional components, and constructed nano ZrO_2_/ZMS composites with different mass ratios by ultrasonic dispersion, which were then mixed with 2.0 wt% SA, physically embedded, and dropped into FeCl_3_ solutions of different concentrations (1–5%, *m*/*v*) for ionic crosslinking to prepare nano ZrO_2_/ZMS composite microspheres. The adsorption study of nitrate indicated that the optimal performance was achieved at a ZrO_2_/ZMS mass ratio of 1:8 and an FeCl_3_ concentration of 3%, with a maximum adsorption capacity of 55.42 mg/g. The SEM results showed that the microspheres were overall regular spherical in shape, with a rough surface and irregular pore structures distributed, which was conducive to providing abundant adsorption sites. BET analysis further confirmed that the material had a high specific surface area (142.62 m^2^/g) and mesoporous structure (average pore diameter 3.82 nm). Since the radius of the nitrate ion (0.34 nm) was much smaller than the pore size, it could smoothly enter the pore channels and undergo effective adsorption. FTIR results indicated that the characteristic functional groups of SA, ZrO_2_, and ZMS were simultaneously present in the composites, indicating successful multi-component compositing. Vibrational peaks corresponding to Zr–O, Si–O–Si, and Si–O–Al demonstrated the integrity of the inorganic framework, and the newly emerged Fe–O/Fe–OH peaks indicated the successful introduction of iron oxides into the system. At the same time, the carboxyl peak of SA shifted and partially disappeared, indicating that Fe^3+^ coordinated and crosslinked with SA, thereby constructing a structurally stable composite microsphere. Swistun et al. [66] used fly ash as the raw material and synthesized NaX-UP type zeolite through the alkali fusion–hydrothermal method, and then modified it with polyethyleneimine (PEI) by amination. Under acidic conditions, the protonated amino groups (e.g., –NH^−^ and –NH_2_) could adsorb anions through hydrogen bonding and electrostatic interactions. Subsequently, Fe_3_O_4_ magnetic nanoparticles were in situ loaded onto the surface, endowing the material with good magnetic responsiveness. Finally, the composite was then mixed with a 2% (*w*/*v*) sodium alginate solution and dropped into a 3% (*w*/*v*) CaCl_2_ solution to form crosslinked microspheres. The characterization results of BET, XRD, and SEM–EDS proved that the material had a high specific surface area, good crystallinity and uniformly dispersed magnetic particles, with abundant active sites such as amino and hydroxyl groups on the surface.

Bentonite is a typical layered silicate mineral with a high specific surface area, good swelling capacity and significant cation exchange capacity. Its interlayer structure can provide abundant adsorption sites for pollutants. In a ternary composite system, the introduction of bentonite can effectively increase the specific surface area and adsorption capacity of the material, and its layered structure is conducive to the construction of a multi-level pore structure, enhancing the mass transfer performance. Moreover, the negatively charged surface of bentonite enables electrostatic interactions with metal oxides and alginate, thereby improving the interfacial bonding and dispersion of components [67]. The bentonite/metal oxide/alginate system is usually suitable for stepwise composite processes, such as physical mixing–ionic crosslinking with in situ loading, to construct microsphere materials. Chkirida et al. [68] used natural bentonite as the inorganic framework, sodium alginate as the polymeric gel matrix, and prepared TiO_2_/bentonite/alginate (TiO_2_-Bnt-Alg) composite microspheres by a stepwise composite approach. Firstly, bentonite was uniformly dispersed in a 2.5% (*w*/*v*) SA, and then the mixture was dropped into a mixed solution containing 2.5% (*w*/*v*) CaCl_2_ and 0.5% (*w*/*v*) TiO_2_ (P25), achieving microsphere formation through ionic crosslinking and in situ loading of TiO_2_; after aging for 12 h and freeze-drying, a dense and high mechanical strength composite was obtained. FE-SEM results showed that the microspheres were composed of a network structure of polymer fibers and nodes, with bentonite uniformly dispersed in a honeycomb-like matrix, having swelling properties. The microsphere surface was rough with wrinkles and protrusions, which were conducive to providing active sites and enhancing pollutant capture ability. TiO_2_ nanoparticles were uniformly dispersed without obvious agglomeration, attributed to the reasonable impregnation process. TGA results indicated that the introduction of bentonite significantly improved the thermal stability of alginate-based microspheres, with the total mass loss reduced from approximately 88% to 52.1%, indicating that bentonite played a role of structural support and thermal insulation in the system, delaying the thermal degradation process of the polymer. The leaching experiment showed, after five consecutive cycles of TiO_2_-Bnt-Alg, only a very small amount of TiO_2_ (≤2 ppm) was released, with no obvious leaching, indicating that the nanoparticles were well immobilized in the biopolymer matrix and exhibited a stable electrostatic interaction with bentonite, thereby effectively enhancing the structural stability and resistance to material loss. The SA/bentonite/TiO_2_ composite microsphere has a significant adsorption–photocatalysis synergistic effect. Pollutants are first rapidly enriched by adsorption, and then undergo deep mineralization through photocatalysis, thereby achieving efficient removal. Within 1 h, a 98% decolorization rate and a 93% COD removal rate can be achieved. Compared with a single adsorption system, this composite material not only significantly improves the removal efficiency but also accelerates the reaction rate and enhances the thoroughness of pollutant degradation.

Introducing inorganic non-metallic framework materials into the SA/metal oxide binary composite system can significantly enhance the material performance through both structural regulation and interfacial optimization. Represented by SiO_2_ and MCM-41, possess a high specific surface area, an adjustable pore structure, and abundant Si–OH. The materials can not only serve as structural supports and dispersion carriers, effectively inhibiting the agglomeration of metal oxide nanoparticles, but also enhance the interface bonding through hydrogen bonds or electrostatic interactions, thereby improving the mechanical strength, structural stability, and utilization efficiency of active sites. Meanwhile, the introduction of SiO_2_ coating or mesoporous structure facilitates the stable immobilization and uniform distribution of metal oxide particles, reducing the risk of metal ion leaching. Zeolite, relying on its regular pore structure and ion exchange properties, not only enhances the structural stability in the ternary system as a rigid framework, but also achieves selective enrichment of target pollutants through pore sieving and ion exchange mechanisms. The synergistic interactions between the surface active sites and metal oxides contribute to improving the dispersion of active components and enhancing adsorption capability. Bentonite, as a layered silicate mineral, utilizes its high specific surface area, interlayer structure, and negative charge to construct a multi-level pore structure, increasing the specific surface area and mass transfer performance. In addition, electrostatic interactions promote the uniform distribution of metal oxides in the matrix and effectively inhibit their leaching, thereby significantly improving the structural stability and reusability of the material. In terms of preparation methods, inorganic non-metallic frameworks can be introduced through one-step methods such as blending, self-assembly or ionic crosslinking, to achieve uniform dispersion and simultaneous formation, or through a stepwise composite strategy to further optimize the structure and functional distribution. The introduction of inorganic non-metallic frameworks significantly improves the structural compactness, surface roughness, thermal stability, and reusability of the microspheres, while ensuring the fixation and uniform distribution of metal oxides, thereby constructing a functional microsphere system with high adsorption capacity, excellent stability, and reusability.

#### 2.2.2. Ternary Systems Based on Metal Nanoparticles

Adding metal nanoparticles as the third component to the SA/metal oxide binary composite system has become an effective strategy for enhancing the functionality of materials. Metal nanoparticles can not only endow composite systems with catalytic activity, antibacterial properties or magnetic functions, but also improve performance through electron transfer or synergistic effects. Common preparation methods include one-step composite processes, such as ionic crosslinking and the in situ reduction method. As well as stepwise composite processes, including the physical encapsulation—in situ reduction method, ionic crosslinking—in situ loading method. These methods can achieve functionalized loading of metal components while maintaining the structural integrity of the alginate matrix, thereby constructing high-performance ternary composite systems.

Ghorbani-Vaghe et al. [69] prepared Fe_3_O_4_@Alg-Au NPs composites by a stepwise composite strategy. Firstly, Fe_3_O_4_ nanoparticles were synthesized by a hydrothermal method and then dispersed by ultrasonic treatment to form Fe_3_O_4_@Alg with SA. Subsequently, HAuCl_4_ solution was introduced to supply Au^3+^, and then reduced by sodium citrate to in situ generate Au nanoparticles in the matrix, yielding Fe_3_O_4_@Alg-Au NPs. Characterization results by FTIR and XRD indicated that Au nanoparticles were successfully loaded onto the Fe_3_O_4_@Alg complex. FE-SEM and TEM results demonstrated that the material had an overall quasi-cubic morphology, with Fe_3_O_4_ particle sizes ranging from 150 to 250 nm and Au nanoparticle sizes from 10 to 15 nm. A uniform SA coating layer was present on the surface of Fe_3_O_4_, on which Au nanoparticles were stably dispersed and immobilized, although slight aggregation was still present. In terms of performance, Au nanoparticles served as the catalytic active center, effectively promoting electron transfer and reduction reactions, while the Fe_3_O_4_@Alg structure provided a stable loading and anchoring environment for Au. The composite was applied for the catalytic reduction of 4-nitrophenol (4-NP) to 4-aminophenol (4-AP). The catalytic reaction results showed that, under room temperature aqueous conditions, only a very low catalyst dosage (1–3 mg) enabled up to 99% conversion within 1.5–4 min. The composite maintained high catalytic activity after eight reuse cycles, indicating good stability and reusability. Vincekovi’c et al. [70] prepared AgNPs-ZnONPs by the ionic gelation method; FTIR confirmed the existence of hydrogen bonding and coordination interactions between nanoparticles and the polymer matrix. AgNPs contributed to a looser network structure, presenting high porosity and good swelling properties, while ZnONPs enhanced compactness of the matrix through the COO^−^ and Zn^2+^ synergistic effect. EDS analysis indicated that Zn was enriched on the surface of the composite, and Ag was mainly distributed inside the microspheres; SEM results showed that the surface of Ag-loaded particles was rough and porous, while ZnO-loaded particles were relatively smooth, and co-loading achieved a balance in structure and release kinetics when both were co-loaded. Antifungal tests showed that the AgNP alginate microspheres had an inhibition rate of 80.7%, 91.4% for ZnONP-alginate microspheres, and the AgNP-ZnONP alginate microspheres had an inhibition rate as high as 99.7%. Microscopic observations confirmed that the co-loaded samples could cause damage to the fungal cell membrane, hyphal collapse, and reactive oxygen species (ROS)-induced injury, exhibiting the strongest antifungal effect, but there were still limitations, such as restricted Ag loading rate in coexistence and the difficulty in establishing a precise release model due to the complex pore structure.

Introducing metal nanoparticles as the third component can significantly enhance the functionality of the composites, while the SA matrix structure can provide stable and dispersed support for metal nanoparticles, ensuring the stability of the material during multiple cycles. However, challenges such as metal nanoparticle aggregation and limited loading capacity still exist. Therefore, in the design of ternary composites, it is essential to balance performance enhancement and structural controllability.

#### 2.2.3. Ternary Systems Based on Natural Polymer Materials

Based on the binary SA/metal oxide system, another type of natural polymer material is introduced to construct ternary composites, which can form hydrogen bonding or electrostatic interactions with alginate molecular chains and coordinate interactions with the surface of metal oxides, thereby constructing a more stable and multi-level network structure in the system. In terms of synthesis methods, the blending–ionic crosslinking method is usually adopted. Chitosan or cellulose is mixed with the SA solution in a certain ratio, and then pre-prepared or in situ generated metal oxide nanoparticles are introduced. Subsequently, a composite gel or microsphere is formed through crosslinking with Ca^2+^ or other multivalent ions.

Chitosan is a typical amino polysaccharide, featuring wide availability, environmental friendliness, and good chemical stability, and is often used as a structural control component in composites. The amino groups (–NH_2_) on the chitosan molecule chain are prone to protonation under acidic conditions to form –NH_3_^+^, which can interact electrostatically with the –COO^−^ in alginate, thereby enhancing the compactness and stability of the network structure. Additionally, this property also endows the material with good adsorption capacity for anionic pollutants. Moreover, SA is prone to water absorption, swelling and even structural damage under alkaline conditions, resulting in a decrease in mechanical strength. While introducing chitosan, the –NH_2_ exist in a deprotonated form, making the molecular chain more rigid and less soluble, which contributes to improved anti-swelling ability and mechanical stability. Therefore, the introduction of chitosan enables the composite system to achieve stable application over a wider pH range.

Zeng et al. [71] utilized waste iron sludge from a water treatment plant as the iron source, prepared Fe_3_O_4_ nanoparticles by the co-precipitation method, and mixed them with iron sludge powder at a mass ratio of 1:3, then dispersed them into a 2.0% (*w*/*v*) SA and 4.67% (*w*/*v*) chitosan mixed solution. To promote the electrostatic interaction between sodium alginate and chitosan, 10 mL of 5% (*v*/*v*) HCl was added to the mixture and stirred. After standing for degassing, the solution was dropped into a 1% (*w*/*v*) CaCl_2_ solution to form microspheres via ionic crosslinking, finally obtaining magnetized sodium alginate–chitosan porous microspheres (M-ACFBs) through vacuum freeze-drying for 8 h at –50 °C. Under acidic conditions, the –NH_2_ in chitosan is protonated to –NH_3_^+^, which forms a stable double-gel network through electrostatic interaction with the –COO^−^ in alginate. The freeze-drying process converts the hydrogel into an aerogel, retaining the three-dimensional polymer network with regular pores, significantly increasing the specific surface area, and providing more sites for As (V) adsorption. The magnetic nanoparticles and iron sludge are fixed in this network and uniformly dispersed, effectively avoiding nanoparticle agglomeration and reducing Fe_3_O_4_ oxidation under the coating of sodium alginate and chitosan. SEM, BET, and VSM characterization shows that the microspheres have a uniform particle size of approximately 2 mm, with a rough surface and grooves. The specific surface area reaches 115.4 m^2^/g, and an average pore diameter of 5.76 nm. Plate-like Fe_3_O_4_ crystals with a side length of approximately 5 μm indicate that the adsorbent has successfully endowed with magnetic properties, with a saturated magnetization of 15.0 emu/g. M-ACFBs present a three-dimensional network, mainly attributed to the framework support of SA and chitosan and the unique freeze-drying method that retains the morphology and structure of the microspheres. FTIR analysis shows that –OH/–NH_2_ (3443 cm^−1^), –COOH (1632 and 1415 cm^−1^), Fe–O (580 cm^−1^), and a small amount of Si–O–Si (1003 cm^−1^) can all be detected, indicating that the structure of SA, chitosan, and magnetic components in the microspheres is uniform and chemically stable. The microspheres are structurally stable within the pH range of 2 to 10 and have low iron leaching.

Cellulose and its derivatives have high crystallinity and abundant -OH, which can form a stable three-dimensional network skeleton with SA through hydrogen bonding, thereby significantly enhancing the mechanical strength and structural stability of composites. Meanwhile, cellulose can act as a dispersing agent to disperse metal oxide nanoparticles. Gong et al. [72] prepared SA/CMC/MnO_2_/Fe_3_O_4_ magnetic microspheres with 1.67% (*w*/*v*) SA as the framework and 0.5% (*w*/*v*) carboxymethyl cellulose as the dispersant, followed by uniformly compounded MnO_2_ and Fe_3_O_4_. The mixed solution was then dropped into a 3.0 wt% CaCl_2_ solution for ionic crosslinking and freeze-drying. FTIR confirmed the presence of Mn-O and Fe-O bonds in the microspheres, along with two characteristic peaks located at 1430 cm^−1^ and 897 cm^−1^, which were formed by the –C–O–C– functional groups and –CH_2_ vibrations of cellulose and sodium alginate, respectively. SEM shows that the material has a honeycomb-like open porous structure inside, with pore diameters ranging from 200 to 300 μm. SEM magnified images show that there are many protruding nanoparticles embedded within the matrix. Upon further magnification, the MnO_2_/Fe_3_O_4_ clusters are uniformly distributed; BET indicates that the composite exhibits a mesoporous–macroporous structure, with an average pore size of 23.57 nm, a specific surface area of 3.06 m^2^/g, and a pore volume of 0.018 cm^3^/g. A porous structure is formed in the composite, which is conducive to ion adsorption.

By introducing chitosan and cellulose and their derivatives, the SA/metal oxide composite system can be synergistically optimized from both molecular interaction and structural construction. Chitosan enhances the compactness and pH adaptability of the gel network through electrostatic interaction and simultaneously improves the mechanical stability of the composite. Cellulose-based components enhance the mechanical strength of the material and promote the uniform dispersion of nanoparticles through hydrogen bonding, constructing a multi-level pore structure, increasing the specific surface area and active sites, thereby significantly improving the application stability of the material in complex water environments.

#### 2.2.4. Ternary Systems Based on Porous Carbon-Based Materials

Porous carbon-based materials (such as activated carbon, biochar, and graphene) typically possess high specific surface area, abundant pore structures, and good chemical stability, which can provide more active sites for the composite and improve mass transfer performance. Meanwhile, the oxygen-containing functional groups (such as –OH and –COOH) on their surfaces can form hydrogen bonding or electrostatic interactions with the alginate matrix, thereby enhancing the interfacial bonding and improving structural stability. Biftu et al. [73] prepared activated carbon (ACLEA) from Epipremnum aureum leaves through concentrated sulfuric acid activation, and synthesized nano-cerium oxide (nCeO_2_) using green extracts of Sapindus mukorossi. After compositing 2% (*w*/*v*) ACLEA with 1% (*w*/*v*) CeO_2_, the mixture was embedded in 2.5% (*w*/*v*) alginate, and dropped into a ZrOCl_4_·8H_2_O crosslinking agent to prepare nCeO_2_@ACLEA-Zr-Alg microsphere adsorbents. XRD results indicated that the prepared material contained nano-CeO_2_ with a typical fluorite crystal structure, along with the characteristic peaks of the graphite microcrystalline structure of activated carbon and the diffraction peaks of Zr-Alg, suggesting that no phase destruction occurred during the composite process and indicating the coexistence of multiple components. FTIR analysis revealed the presence of abundant surface functional groups in the material, including –OH, C–H, C=O, C–O–C, as well as vibration peaks of Zr–O and Ce–O. The existence of these functional groups provided potential active sites for the material, which are beneficial for the binding and adsorption of pollutants. EDS results showed that the main elements in the composites included C, O, Ce, and Zr, indicating the successful combination of activated carbon, CeO_2_, and Zr-Alg. FESEM revealed that the surface of the composite microspheres presented a rough, porous, and sponge-like structure, which was conducive to providing a large specific surface area and more adsorption sites.

Albqmi et al. [74] successfully constructed magnetic nanocomposite hydrogel microspheres using alginate (Alg), graphene oxide (GO), and CoMnFeO_4_ magnetic nanoparticles as raw materials through an ionic crosslinking method (Figure 4b). Firstly, under alkaline conditions (pH ≈ 11), CoMnFeO_4_ nanoparticles were synthesized by co-precipitation with Mn^2+^, Co^2+^, and Fe^3+^ at a molar ratio of 1:1:2. Meanwhile, GO was prepared by an improved Hummers method, introducing abundant oxygen-containing functional groups to enhance the surface activity of the material. Subsequently, 15% (*w*/*v*) CoMnFeO_4_ nanoparticles and 20% (*w*/*v*) GO were dispersed in SA solution, and the mixed solution was dropped into 1 M CaCl_2_ solution (40 mL) to prepare Alg–GO–CoMnFeO_4_ hydrogel microspheres. In terms of material characterization, FTIR analysis indicated that-COOH and -OH were oxygen-containing functional groups of GO. The metal–oxygen bond of CoMnFeO_4_ was simultaneously present in the composite, and some peak shifted, suggesting the existence of hydrogen bonds and interactions among the components. XRD results showed that the typical spinel crystal phase characteristic peaks of CoMnFeO_4_ were retained in the material, while the diffraction peaks of GO weakened, indicating its effective dispersion and embedding in the matrix. SEM observations revealed that the composite microspheres had a rough and porous structure, with nanoparticles uniformly distributed on both the surface and within the interior, which was beneficial for increasing the specific surface area and mass transfer performance. BET analysis results indicated that the specific surface area was 65.9778 m^2^/g, and the pore size distribution was 4.75–6.13 nm, presenting a typical mesoporous structure, which was conducive to the diffusion and adsorption of pollutants. VSM tests showed that the saturation magnetization of the composite was approximately 18.6 emu/g, demonstrating good magnetic responsiveness and enabling rapid solid–liquid separation. In addition, TGA further confirmed that the material had good thermal stability, and the introduction of inorganic components significantly improved the structural stability of the system. The composite exhibited remarkable adsorption properties for dimethoate, with a maximum adsorption capacity of 196 mg/g and a removal rate of 99.28%. After six adsorption–desorption cycles, the removal efficiency remained above 80%, indicating good reusability.

Wang et al. [75] used SA and Mg/Fe bimetallic oxide-modified biochar (MFBC) as the main components and employed the ionic crosslinking method to construct magnetic SA@MFBC aerogel microspheres. Firstly, the modified biochar (MFBC) loaded with MgO and Fe_3_O_4_ was prepared through hydrothermal reaction and high-temperature carbonization. Then, 6% (*w*/*v*) MFBC and 3% (*w*/*v*) SA were mixed and dropped into a 1% (*w*/*v*) CaCl_2_ solution to form the microspheres. Finally, the aerogel microspheres with a three-dimensional porous structure were obtained through freeze-drying. SEM showed that the microsphere diameter was approximately 1.5 mm, with a rough droplet-like surface, and the protruding parts of the surface had obvious pores. The interior of the microspheres was a three-dimensional porous aerogel structure. The internal and external channels and pore structure provided transport channels and more adsorption sites. BET analysis showed an average pore diameter of 3.41 nm, mainly consisting of micropores and mesopores, with a specific surface area of 122.48 m^2^/g, which might be due to the introduction of magnesium, promoting the expansion of biochar pores and the formation of new pores. TGA results indicated that the thermal stability of the microspheres significantly improved after the introduction of MFBC. VSM showed a magnetic response of 20.43 emu/g, indicating good magnetic responsiveness and enabling rapid magnetic separation. XPS and FTIR further confirmed the participation of –COOH, –OH, and metal–oxygen bonds in the adsorption process. SA@MFBC had a high adsorption capacity for U(VI), reaching 545.35 mg/g. After five adsorption–desorption cycles, more than 80% of the adsorption capacity was retained, indicating superior stability and reusability. Functionally, biochar served as the framework and adsorbent, providing abundant pore structures and surface functional groups, and significantly enhancing the adsorption capacity by loading metal oxides, while improving the mechanical properties and stability of the composite. It is a key component for achieving high-capacity adsorption and structural reinforcement.

In the construction of alginate-based composite adsorbents, SA/metal oxides have been widely studied, and their synthesis methods are mainly categorized into ionic crosslinking and in situ synthesis methods. Among them, the ionic crosslinking method has a simple process and mild conditions, making it easy to achieve large-scale preparation. However, since the interaction between nanoparticles and the matrix mainly relies on physical interaction, the interfacial binding is relatively weak, and the reusability is insufficient, leading to particle agglomeration or loss. In contrast, the in situ synthesis method enables the in situ generation of metal oxides within the matrix, enhancing the interfacial bonding and dispersion. However, this method usually has higher requirements for the control of reaction conditions, and the controllability and universality of the system are still limited. Moreover, it mostly focuses on the construction of binary structures and has deficiencies in the coordinated regulation of multiple components.

Although the SA/metal oxide binary composite system has significantly improved in structural stability and functional synergy compared to pure alginate materials, it still has limitations such as easy agglomeration and leaching of metal oxides, low specific surface area, single pore structure, and insufficient interfacial binding, which limit the utilization efficiency of active sites and the reusability. Therefore, introducing a third component to construct a ternary composite system has become an important strategy for further optimizing material performance. From the perspective of the synthesis method, the ternary system is still primarily based on the ionic crosslinking method and mostly adopts the combination strategies of “blending–ionic crosslinking” or “in situ blending–ionic crosslinking”. Only a few studies involve multi-step synthesis processes to achieve fine structural regulation. On this basis, the third component compensates for the limitations of the binary system through structural regulation, interface interaction, and multi-functional synergy. On the one hand, by providing a higher specific surface area, constructing a multi-level pore structure, and enhancing the framework, it effectively promotes the uniform dispersion of metal oxides, reduces particle agglomeration, and increases the utilization of active sites. On the other hand, electrostatic interactions, hydrogen bonding or coordination among multiple components enhance interfacial bonding, which inhibits the leaching of metal ions and the loss of active components, and significantly improves the mechanical stability and reusability. Meanwhile, certain functional components can also achieve selective enrichment of target pollutants through pore-size sieving, ion exchange and surface charge regulation, and further expand the multi-functionality in adsorption, catalysis, antibacterial and magnetic separation. The ternary composite system has achieved a systematic breakthrough in the limitations of the binary system through a synergistic mechanism of structural optimization, interface enhancement and functional expansion.

All of these synthesis strategies have been successfully employed to fabricate alginate-based ternary composites; each approach exhibits distinct characteristics in terms of interfacial integration, structural homogeneity, and preparation complexity. Physical mixing is straightforward and cost-effective but generally results in relatively weak interfacial interactions. In contrast, self-assembly and in situ growth facilitate stronger interfacial coupling and more uniform distribution of functional components, although they usually require more stringent control over synthesis conditions. The sol–gel method is particularly suitable for constructing homogeneous inorganic frameworks but involves relatively complex preparation procedures. Therefore, no single synthesis strategy is universally superior, and the optimal method should be selected according to the desired composite structure, target pollutants, and practical application requirements. The main advantages and limitations of different synthesis strategies are summarized in Table 2.

## 3. Applications of Alginate-Based Composites to Wastewater Treatment

### 3.1. Removal of Heavy Metals

Heavy metals are generally defined as metallic elements with relatively high density that can exert toxicity even at trace levels. Due to their strong toxicity, non-biodegradability, and easy accumulation in organisms, they have become an important challenge in the field of water environment [76]. Common heavy metal ions such as Pb^2+^, Cd^2+^, Cu^2+^, Ni^2+^, As(V), and Cr(VI) are widely derived from industrial wastewater in electroplating, metallurgy, mining, and chemical industries. Their long-term existence poses a serious threat to the ecosystem and human health [77]. Ternary composites have shown good application potential in the field of heavy metal removal due to their advantages of multi-component synergy.

In the ternary system, inorganic non-metallic frameworks mainly function through structural regulation and interface synergy. On the one hand, their high specific surface area and porous structure can provide abundant adsorption sites and promote mass transfer. On the other hand, functional groups such as –Si–OH and –Al–OH on the surface can coordinate with heavy metal ions, enhancing chemical adsorption. Meanwhile, their structural support and confinement effect can inhibit nanoparticle agglomeration, improve dispersion and stability, and achieve synergistic enhancement of heavy metal removal performance through multiple interface interactions.

Safari et al. [78] prepared Fe_3_O_4_@bio-SiO_2_/Alg (MBA) by co-precipitation combined with ionic crosslinking. In this system, Fe_3_O_4_ provides a magnetic response for rapid separation, while bio-SiO2 endows a high specific surface area and abundant Si–OH, and effectively inhibits nanoparticle agglomeration and enhances structural stability. Alginate serves as the biological scaffold, providing –COO^−^ and –OH, which can act as a fixation matrix to prevent nanoparticle loss and enhance adsorption capacity and reusability. Under optimal conditions (pH 7, 180 min, 1.0 g/L, 40 mg/L), the material achieved a Cd(II) removal efficiency of 79.23%, with a maximum adsorption capacity of 35.36 mg/g, and still maintained 52.6% after five cycles. Mechanism analysis indicates that multiple synergistic effects mainly drive the adsorption process: (i) coordination complexation between surface functional groups (–COO^−^, –OH, and Si–OH) and Cd^2+^; (ii) electrostatic attraction induced by the negative charge on the material surface under neutral conditions; and (iii) the physical confinement and site enrichment effect provided by the porous structure. Kinetic and isothermal model fitting results indicate that this process follows pseudo-second-order kinetics and a Langmuir isotherm, suggesting a spontaneous exothermic process dominated by monolayer chemisorption.

Metallic materials can provide active sites and impart multi-functionality to the materials in the ternary system. Jawed et al. [79] constructed NH_2_-Fe(III)/ZnO/calcium alginate ternary composite microspheres (FZO-M) by the ionic crosslinking method. In this system, Fe(III)-ZnO served as the core adsorption component, providing a high specific surface area and abundant surface hydroxyl sites. The formation of the CuO layer through the interaction of surface hydroxyl groups with Cu(II) was the main removal mechanism. APTES amine modification introduced –NH_2_ functional groups to enhance surface coordination ability and electrostatic adsorption. SA served as the three-dimensional polymer framework, achieving stable fixation of inorganic components through -COOH and -OH, while enhancing adsorption capacity, mechanical stability, dispersibility, and reusability. Under optimized conditions (pH 4, 25 °C, 1 g/L, 12 h), the removal rate of 50 mg/L Cu(II) reached 98.25%, and the maximum adsorption capacity was as high as 2144.5 mg/g. The kinetic results conformed to the pseudo-second-order model, indicating that chemisorption dominates the adsorption process. The isotherm conformed to the Langmuir model, indicating that Cu(II) was mainly adsorbed on the material surface as a monolayer adsorption. Thermodynamic analysis indicated that the adsorption was an exothermic process. Mechanism analysis indicated that the removal of Cu(II) by this ternary system was driven by multiple synergistic mechanisms. Firstly, under acidic conditions, the -NH_2_ on the material surface were protonated to form -NH_3_^+^, promoting the enrichment of Cu(II) on the material surface through electrostatic interaction. Subsequently, coordination complexation between –NH_2_/–NH_3_^+^ groups and Cu(II) serves as the primary source of chemical adsorption. Meanwhile, the Zn-OH/Fe-OH sites on ZnO and Fe(III) participated in the reaction, promoting the formation of Cu-O bonds and inducing surface deposition of CuO/Cu_2_O, thereby further strengthening the fixation effect. Furthermore, the –OH/COOH groups in the alginate network also participated in adsorption through hydrogen bonds and auxiliary coordination, stabilizing the structure. Overall, this system achieved a static enrichment, amine complexation, metal oxide deposition multi-mechanism coupling process, significantly enhancing the removal efficiency of Cu(II). Overall, the synergistic integration of multiple mechanisms leads to a significant enhancement in the removal efficiency of Cu(II).

**Figure 5:**
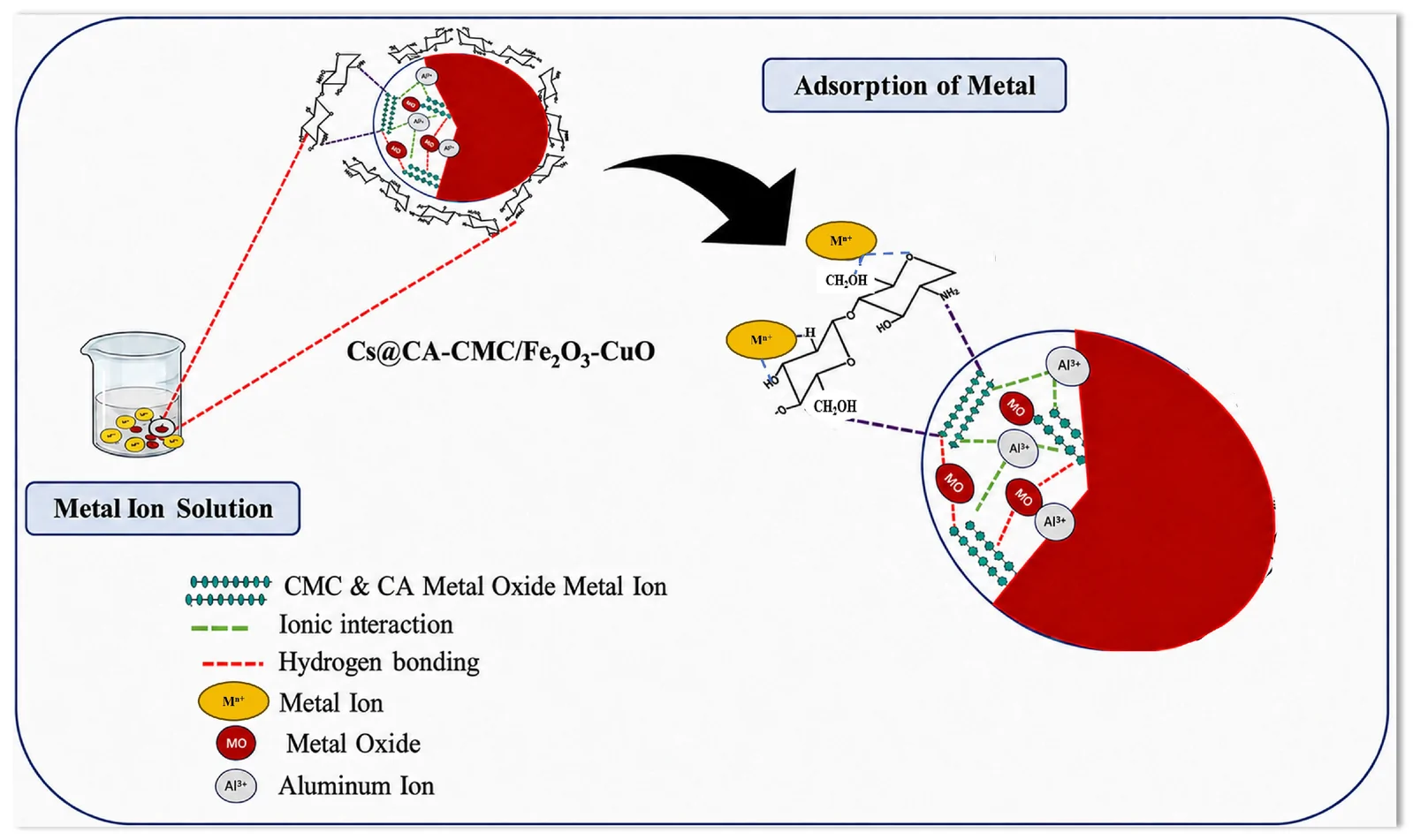
Proposed mechanism of metal ion adsorption onto Cs@CA-CMC/Fe_2_O_3_-CuO beads. Reprinted from Maslamani et al. [81]. Copyright (2024), with permission from Taylor & Francis Group, LLC.

Natural polymers, as the third component, possess both structural regulation and adsorption enhancement functions. On the one hand, they form a three-dimensional gel network with SA, which can improve structural stability and promote mass transfer. On the other hand, the functional groups such as –COO^−^, –NH_2_, and –OH on the material surface can combine with heavy metal ions through electrostatic attraction, ion exchange, and coordination complexation, thereby providing abundant active sites and enhancing adsorption capacity and selectivity. Gong et al. [80] prepared SA/CMC/MnO_2_/Fe_3_O_4_ magnetic porous microspheres by ionic crosslinking combined with freeze-drying. The material has an equilibrium adsorption capacity of 38.8 mg/g for Tl^+^, with residual thallium concentration in the effluent reduced to below 0.1 μg/L. The adsorption kinetics conforms to the pseudo-second-order model, indicating that the process is mainly chemisorption. The process also conforms to the three-stage intraparticle diffusion model, including surface diffusion, pore internal diffusion, and the adsorption equilibrium stage, and internal diffusion is not the sole rate-controlling step. The adsorption isotherm is better described by the Freundlich model (maximum capacity 50.20 mg/g), indicating a multilayer adsorption behavior. Thermodynamic analysis shows that this process is an exothermic spontaneous process (ΔH = 21.20 kJ/mol, ΔG < 0, ΔS > 0), and the adsorption capacity increases with temperature. The material maintains a high removal efficiency within the pH range of 2–9 and shows significant selectivity for Tl^+^, with selectivity coefficients as high as 10^4^ compared to other heavy metal ions such as Cu^2+^, Cd^2+^, and Pb^2+^. After five cycles, the removal rate is still higher than 83.9%, demonstrating good stability. In terms of the adsorption mechanism, redox reactions and ion exchange play a dominant role. MnO_2_ oxidizes Tl^+^ to Tl^3+^ (Mn^4+^ is reduced to Mn^2+^) through redox action, while Fe_3_O_4_ surface hydroxyl participates in H^+^/Tl^+^ ion exchange, and the SA/CMC three-dimensional porous structure provides mass transfer channels and structural support for adsorption. In this system, CMC acts as an auxiliary polymer component, jointly constructing a composite gel network with SA. It can regulate the pore structure and mechanical stability of the microspheres and enhance the dispersion and fixation of active components through its abundant carboxyl groups, thereby promoting the uniform distribution of MnO_2_ and Fe_3_O_4_ in the matrix and indirectly improving the overall adsorption properties of the material. The synergistic interactions among these components endow the ternary composite with good overall performance in heavy metal removal. Maslamani synthesized Fe_2_O_3_-CuO bimetallic oxide by co-precipitation and mixed it with calcium alginate and carboxymethyl cellulose before ionic crosslinking with AlCl_3_ to form microspheres, which were finally coated with chitosan to prepare Cs@CA-CMC/Fe_2_O_3_-CuO composite microspheres [81]. The microsphere shows good adsorption properties for Ag(I), with an adsorption capacity of 4.91 mg/g and a removal rate of 98.12%. The adsorption process conforms to the pseudo-second-order kinetic model and the Langmuir isothermal model, with a theoretical maximum adsorption capacity of 10.12 mg/g, indicating that monolayer chemisorption is dominant. The adsorption mechanism (Figure 5) is mainly dominated by the coordination and electrostatic attraction between the –NH_2_ and –OH functional groups in chitosan and Ag(I). Meanwhile, the –COO^−^ in carboxymethyl cellulose (CMC) provide binding sites through electrostatic interaction and ion exchange. The hydroxyl groups on the surface of Fe_2_O_3_–CuO participate in coordination complexation, further enhancing the adsorption capacity. Additionally, the three-dimensional gel network constructed by calcium alginate and CMC can form a stable porous structure, increase the specific surface area, and promote the diffusion of metal ions into the material and their participation in the ion exchange process, thereby achieving efficient capture of heavy metal ions through the synergistic effect of multiple mechanisms.

Porous carbon-based materials mainly function as high specific surface area functional frameworks in the ternary system, providing abundant oxygen-containing functional groups that promote the efficient removal of heavy metal ions through electrostatic adsorption and surface complexation [82]. Luo et al. [83] prepared magnetic alginate sodium composite microspheres BC/MCAC by the ionic crosslinking method. This material exhibited a good adsorption effect over a wide pH range, with the maximum adsorption capacity for Pb(II) being 211.6 mg/g. The adsorption process conformed to the Langmuir isothermal model and pseudo-second-order kinetics, being spontaneous and endothermic monolayer chemisorption. After three cycles, it still retained 82.37% of removal efficiency.

In this system, biochar (BC) served as the main carbon-based framework, with its surface rich in oxygen-containing functional groups such as –COOH and –OH, which could enrich Pb^2+^ through electrostatic interaction and form complexation with it, being an important source of chemical adsorption sites. γ-Fe_2_O_3_ not only provides magnetic separation performance but also provides Fe–OH groups that participate in surface complexation with Pb^2+^, enhancing the interfacial activity and dispersion. The calcium alginate network could undergo an ion exchange reaction between Pb^2+^ and Ca^2+^ during adsorption, thereby achieving fixation of Pb^2+^. Malitha et al. [84] prepared magnetic graphene oxide/alginate calcium composite microspheres (MGO@CA) via the improved Hummers method, in situ co-precipitation method, and ionic crosslinking method for rapid adsorption of Pb^2+^ under optimal conditions (pH = 6, dosage = 0.25 g/L, initial concentration = 50 mg/L). The equilibrium adsorption amount was 195.65 mg/g, and the theoretical maximum adsorption capacity was 270.27 mg/g. The adsorption process also conformed to the Langmuir and the Freundlich models, indicating the existence of monolayer and multilayer adsorption. The adsorption kinetics followed the quasi-second-order kinetics, indicating that chemical adsorption played a dominant role in the rate control, and after five cycles, the removal efficiency is still retained 82.28%, demonstrating excellent stability and reusability. Mechanism analysis showed that the removal of Pb^2+^ by MGO@CA was mainly driven by multiple synergistic mechanisms. Firstly, under suitable pH conditions, the material surface undergoes deprotonation, and the negatively charged functional groups promote the rapid enrichment of Pb^2+^ through electrostatic interaction. Subsequently, oxygen-containing groups such as –COOH and –OH in graphene oxide and calcium alginate form surface complexes with Pb^2+^. Meanwhile, the “egg-box” structure of the calcium alginate network with –COO^−^ and Ca^2+^ can form an ion exchange reaction with Pb^2+^, achieving stable fixation of metal ions. In addition, Fe_3_O_4_ not only provided good magnetic separation performance but also provided auxiliary adsorption sites through surface hydroxyl groups and enhanced interfacial reaction activity. The combined action of the three components made the material exhibit high adsorption capacity and rapid removal ability.

To further systematically investigate the removal mechanism of alginate-based ternary composites for heavy metals, Table 3 comprehensively compares the adsorption capacity, removal efficiency (performance enhancement percentages were calculated using data obtained under identical experimental conditions within the same study), adsorption kinetics, and reusability of multiple materials. Additionally, the functional roles and synergistic mechanisms of the components involved in the removal process are specifically summarized.

Ternary composites demonstrate significant structural advantages and functional synergy in the removal of heavy metals. Through the multi-component design of “matrix framework–functional component–regulatory component”, the materials have been effectively enhanced in terms of specific surface area, porous structure, surface chemistry, and interfacial reactivity. Among them, natural polymers such as alginate construct stable three-dimensional network structures and provide abundant functional groups. Metal oxides/metallic materials provide specific active sites and functions such as magnetic separation, catalysis, antibacterial activity, and redox reactions. Inorganic non-metallic frameworks or porous carbon-based materials enhance the mass transfer process and increase the adsorption sites through high specific surface area and porous structure. Different components are coupled in terms of structural support, active-site provision, and interface regulation.

From a mechanistic perspective, the removal of heavy metal ions by the ternary system usually follows a multi-mechanism synergistic pathway, mainly including electrostatic adsorption, surface complexation, ion exchange, and redox reactions. Electrostatic interaction promotes the migration and enrichment of metal ions to the material surface, surface functional groups (such as –COO^−^, –OH, and –NH_2_) enhance the binding strength through coordination complexation, ion exchange achieves stable fixation, and redox reactions in specific systems change the valence state of metals to further improve the removal efficiency. These mechanisms are synergistically coupled among different components, thereby significantly enhancing the adsorption capacity, selectivity, and stability of the material.

### 3.2. Removal of Dyes

With the development of the industry, organic dyes have been increasingly widely used in textiles, printing and dyeing, leather and medicine, and the discharge of dye wastewater has become an important water environmental issue. Dyes are typically characterized by stable structure, high chromaticity and difficult biodegradability, which not only reduce water transparency, inhibit photosynthesis and increase COD, but also may pose potential hazards to ecosystems and human health [86]. Ternary composites, due to their advantages of multi-component synergy and functional integration, have shown significant potential in dye removal. Alginate constructs with a three-dimensional network skeleton provide structural support and functional groups such as –COO^−^ and –OH, which is conducive to the adsorption, enrichment and mass transfer process of dye molecules. Metal oxides, as core functional components, not only provide surface adsorption sites but also endow materials with functions such as adsorption, photocatalysis, antibacterial properties, and magnetic separation. The third component is regulated based on the characteristics of pollutants, enhancing structural stability, increasing specific surface area, optimizing pore structure or introducing specific functional groups, thereby further improving the comprehensive performance of the material and making it a promising system for the efficient removal of dye wastewater.

When inorganic non-metallic frameworks are introduced as the third component into the ternary composite system for dye wastewater treatment, they mainly play a synergistic effect in structural support, active dispersion, mass transfer optimization, and stability enhancement [87]. On the one hand, their high specific surface area and multi-level pore structure can significantly increase the overall porosity, construct rapid mass transfer and electron migration channels, reduce diffusion resistance and enhance pollutant enrichment capacity, thereby promoting the reaction kinetics process. On the other hand, they can serve as a stable skeleton to uniformly load and disperse active components, effectively inhibit the agglomeration and loss of nanoparticles, and thereby improve the utilization rate of active sites.

Chkirida et al. [68] used an ionic crosslinking method combined with an in situ loading method to disperse bentonite in sodium alginate solution, achieving calcium ionic crosslinking and TiO_2_ (P25) surface loading, and prepared sodium alginate/bentonite/TiO_2_ composite gel microspheres (TiO_2_-Bnt-Alg) with both adsorption and photocatalytic properties for the treatment of methylene blue (MB) in water. The results showed that after 60 min of dark adsorption, the COD removal rates of 170 and 320 mg/L MB reached 84% and 85%, respectively, and the decolorization rates were 94% and 95%. After reaching adsorption equilibrium, photocatalysis was initiated, and within 60 min under ultraviolet light, the decolorization rate of MB reached 98%, which was significantly higher than that of pure TiO_2_ (about 10%), sodium alginate (83%), and Bnt-Alg (88%), and the COD removal rate reached 93%. After five cycles, the TiO_2_ leaching amount of this material was less than 2 ppm, and it remained structurally stable within the pH range of 1–12. The mechanism is the synergistic effect of adsorption and photocatalysis. Sodium alginate builds a three-dimensional porous hydrogel skeleton, endowing the material with high swelling capacity and good adsorption ability, while enhancing structural stability and recyclability. Bentonite, as a clay filler, increases the specific surface area and pore structure, enhances the pollutant enrichment capacity, and cooperates to fix the TiO_2_ active components. TiO_2_ generates electron–hole pairs under UV irradiation, further generating strong oxidative ·OH radicals, which can effectively destroy the chromophoric groups of MB and gradually mineralize organic pollutants into CO_2_ and H_2_O. The three components synergistically integrate to achieve a dual removal path of rapid adsorption enrichment and efficient photocatalytic degradation, thereby significantly improving the overall removal efficiency.

Hachemaoui et al. [63] prepared Fe_3_O_4_@MCM-41 composites by mechanical blending and then prepared magnetic composite microspheres MC@CA by ionic crosslinking for the catalytic reduction in methylene blue (MB) in water. The experimental results showed that MC@CA (dosage 4.8 mg) could completely reduce MB within 5 min and could be reused for five cycles while maintaining high efficiency. The catalytic reduction mechanism involves NaBH_4_, providing a strong reducing environment. Fe_3_O_4_ acts as the catalytic active center, promoting the rapid transfer of electrons between BH_4_^−^ and dye molecules, thereby destroying the conjugated chromophoric system of MB and reducing it to a colorless leuco form. MCM-41 mesoporous SiO_2_, with its high specific surface area and ordered pore structure, uniformly disperses Fe_3_O_4_ nanoparticles and provides a rapid channel for reactant and electron transfer, significantly enhancing the catalytic rate. Sodium alginate, as a three-dimensional crosslinked matrix, immobilizes Fe_3_O_4_@MCM-41 to prevent its loss and reduce metal leaching, and endows the microspheres with a good pore structure and hydrophilicity, facilitating the diffusion of pollutants and magnetic separation. The synergy among these components enables efficient, stable, and recyclable catalytic reduction performance.

**Figure 6:**
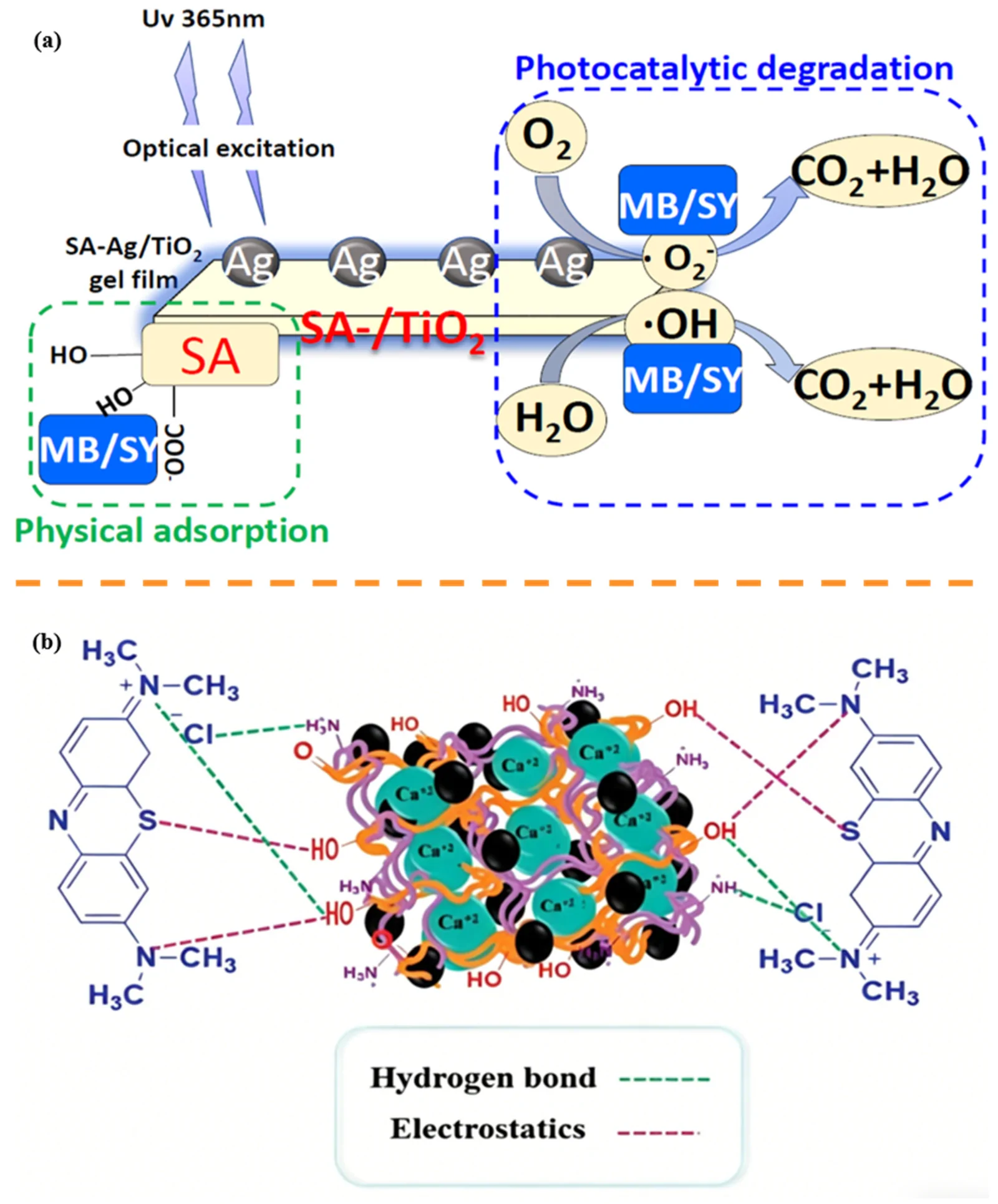
(**a**) The schematic diagrams of physical and photocatalytic pathways for dye removal by the SA-Ag/TiO_2_ gel film. Reprinted from Yang et al. [88]. Copyright (2026), with permission from IOP Publishing Ltd. (**b**) The adsorption mechanism of MB on CS/Al@Fe_3_O_4_ MNCs. Reprinted from Rostami et al. [92]. Copyright (2025), with permission from Wiley Periodicals LLC.

When metallic materials are introduced as the third component into the ternary composite system for dye wastewater treatment, they can be uniformly dispersed through the interface interaction with the matrix and provide more adsorption sites by regulating the microstructure of the material. Specific metal components can offer functions such as photocatalysis, antibacterial activity, and magnetic separation. Yang et al. [88] successfully prepared a SA-Ag/TiO_2_ nanocomposite hydrogel membrane with adsorption–photocatalytic performance using SA as a green reducing and embedding agent through UV reduction and Ca^2+^ ionic crosslinking. The composite demonstrated a strong removal capacity for the cationic dye methylene blue (MB), with a dark adsorption rate of 24.18%, and a photodegradation rate of 45.1%. However, the composite showed a relatively weak removal effect for the anionic dye sunset yellow (SY), with a dark adsorption rate of 2.20%, and a photodegradation rate of 23.8%, indicating a significant selectivity. The degradation process conformed to the pseudo-first-order kinetics, suggesting that the dye concentration mainly controlled the reaction rate. The cycling experiments indicated that the material performance gradually declined with reuse (dropping to 42.9% in the fourth cycle and suddenly to 19.7% in the fifth cycle), mainly due to the gel swelling causing TiO_2_ detachment and the slow destruction of the polymer skeleton by reactive radicals. However, the overall structure still maintained a certain stability. From the perspective of component roles, the Ca^2+^ crosslinked network constructed by SA provided a stable three-dimensional porous skeleton and abundant adsorption sites for the system while functioning as a reducing agent to in situ generate and immobilize Ag nanoparticles. TiO_2_, as the core of photocatalysis, provided the main active sites, generating ·OH and ·O_2_^−^ under UV irradiation to mineralize the dyes. Ag nanoparticles enhance light absorption via surface plasmon resonance (SPR) and promote the separation of photogenerated electron–hole pairs, thereby significantly improving photocatalytic efficiency. In terms of the mechanism, the system followed a synergistic mechanism of adsorption enrichment and photocatalytic degradation. In the dark reaction stage, dye molecules were first enriched on the surface of composites through electrostatic interaction, van der Waals forces, and hydrogen bonding with the –OH and –COOH groups on the SA molecular chain. Cationic dyes were more easily adsorbed due to electrostatic attraction, while anionic dyes were limited in adsorption due to electrostatic repulsion. Under UV irradiation, TiO_2_ generated·OH and ·O_2_^−^, further oxidizing and degrading the dye molecules at the hydrogel–water interface. Meanwhile, Ag nanoparticles attached to the surface of TiO_2_ exhibited the SPR effect, serving as a capture center and transport channel for photogenerated carriers, further enhancing the photocatalytic activity of TiO_2_ (Figure 6a).

Azeroual et al. [89] synthesized Zr/TiO_2_ nanowires (HZTO) by the hydrothermal method and prepared sodium alginate/Zr/TiO_2_ nanowire low-temperature gel microspheres (SA/HZTO) through Ca^2+^ ionic crosslinking combined with freeze-drying. The material exhibited good adsorption properties for methylene blue (MB) and safranin (SF), with adsorption capacities reaching 26 mg/g and 29 mg/g, respectively, and the adsorption process conformed to the pseudo-second-order kinetic model. Thermodynamic analysis indicated that both ΔH° and ΔS° were negative, suggesting that the adsorption was an exothermic process. Within a certain temperature range, ΔG° was negative, indicating that the process was spontaneous and mainly physical adsorption. From the perspective of the mechanism, this system mainly followed an adsorption mechanism dominated by electrostatic interaction and coordinated by multiple interactions. The sodium alginate matrix provides –COOH and –OH active sites and constructs a three-dimensional porous network structure. It preferentially enriches cationic dyes through electrostatic attraction and enhances the adsorption capacity with the assistance of hydrogen bonding and pore-filling effects. Zr/TiO_2_ nanowires further increase the adsorption capacity and enhance the structural stability by increasing the specific surface area and providing additional adsorption sites. The “egg box” structure formed by Ca^2+^ crosslinking effectively improves the mechanical strength and pore integrity. Under the synergistic effect of multiple components, efficient removal of cationic dyes is achieved.

Compared with a single SA matrix, introducing other natural polymers as the third component (such as chitosan, cellulose and its derivatives) can participate in the construction of a denser and more stable three-dimensional network structure through hydrogen bonding or electrostatic interaction between molecular chains. This not only helps to regulate pore size distribution and specific surface area but also enhances the mechanical strength and anti-swelling performance [90]. Meanwhile, natural polymers are rich in functional groups such as -OH, -NH_2_, and -COOH, which can further enrich the types of adsorption sites based on the SA matrix, and promote the uniform dispersion and stable fixation of metal oxides in the matrix. Maslamani et al. [91] used a blending and ion crosslinking method, with AlCl3 as the crosslinking agent, to uniformly disperse CuO–Fe_2_O_3_ nanocomposites in a sodium alginate–carboxymethyl cellulose–chitosan (CA–CMC–CS) polymer matrix, and prepared CuO–Fe_2_O_3_/CA–CMC–CS nanocomposite microspheres. This material exhibited excellent catalytic reduction performance for methyl orange (MO) and eosin yellow (EY) in the presence of NaBH_4_. The removal rate of MO exceeded 94% within about 3 min, and the removal rate of EY reached 97% for 4 min. The corresponding reaction processes conformed to the pseudo-first-order kinetic model, showing a high reaction rate constant. The mechanism is adsorption enrichment coupled with electron transfer catalysis. CuO-Fe_2_O_3_ serves as the catalytic core, providing active sites and electron transfer channels, accelerating the reduction in nitro and azo groups. CA–CMC–CS forms a porous three-dimensional network, adsorbing pollutants through hydrogen bonding, electrostatic interaction, and physical channels, while dispersing nanoparticles to prevent agglomeration, enhance stability and recyclability. The Al^3+^ crosslinking builds a stable structure, ensuring the formation and reusability of the microspheres. This composite microsphere can be recycled three times.

Rostami et al. [92] prepared Fe_3_O_4_ nanoparticles by the co-precipitation method, and then constructed Fe_3_O_4_–chitosan–sodium alginate magnetic nanocomposites (CS/Al@Fe_3_O_4_) by the self-assembly method for the removal of methylene blue (MB) from water. Under optimal conditions (pH = 10, adsorption time of 15 min, dosage of 0.003 g, temperature of 25 °C), the maximum adsorption capacity was as high as 526.32 mg/g, and the adsorption process conformed to the Langmuir isotherm model and pseudo-second-order kinetic model. After three reuse cycles, there was no significant decrease in MB adsorption capacity. The adsorption mechanism is mainly electrostatic interaction and hydrogen bonding. Under alkaline conditions, the material surface is negatively charged, while MB exists in the form of cations, and the electrostatic attraction between the two becomes the dominant force. Additionally, the abundant –NH_2_, –COOH, and –OH in chitosan and sodium alginate can form hydrogen bond networks with dye molecules, further enhancing the adsorption capacity. Fe_3_O_4_ nanoparticles not only endow the magnetic separation performance but also increase the specific surface area and enhance the structural stability. pH has a significant impact on the adsorption process. Under alkaline conditions (pH ≈ 10), the competition of H^+^ weakens and electrostatic attraction strengthens, thereby achieving higher adsorption efficiency (Figure 6b). The synergy of multiple components in terms of structural construction and interfacial interaction enables the material to exhibit high efficiency, rapidity and reusability for MB.

Introducing porous carbon-based materials as the third component (such as activated carbon, biochar, or graphene) can further enhance the removal performance of dyes in terms of structural regulation and interface enhancement. Porous carbon-based materials typically have high specific surface area, well-developed pore structure, and efficient chemical stability, which can provide a large number of additional adsorption sites and promote the enrichment of pollutants on the material surface [93]. Meanwhile, their surfaces are rich in oxygen-containing functional groups (–OH, –COOH) or π-conjugated structures, which can form good interfacial interactions with the SA matrix and metal oxides, thereby improving the dispersion of active components and enhancing the overall structural stability [94]. Additionally, some carbon-based materials also have good electrical conductivity, which is conducive to the electron transfer process and provides support in photocatalysis or reduction reactions. Saning et al. [95] prepared magnetic carbon/sodium alginate composite microspheres (MACBs) using waste iron filings, activated carbon, and sodium alginate as raw materials through ionic crosslinking. The material exhibited excellent removal performance for methylene blue (MB) and Pb(II), with maximum adsorption capacities reaching 476.19 mg/g and 163.93 mg/g, respectively. The adsorption process conformed to the Langmuir isothermal model and the pseudo-second-order kinetic model, and the composite could be rapidly separated under an external magnetic field and maintained a high removal efficiency after multiple cycles. From a mechanism perspective, the system exhibits distinct adsorption mechanisms for different pollutants. For Pb(II), the adsorption is mainly dominated by electrostatic attraction and ion exchange. The developed micropores and mesopores of activated carbon provide sufficient filling space for ions, thereby achieving a high adsorption capacity. For the MB, it is mainly dominated by electrostatic interaction, hydrogen bonds, van der Waals forces, and π–π interactions. The mesoporous structure of activated carbon plays a key role in the adsorption of macromolecular dyes. In terms of the synergy of the three components, SA provides ion exchange and electrostatic binding sites through carboxyl groups, activated carbon contributes a high specific surface area and multi-level pore structure, and waste iron filings provide magnetic response performance and auxiliary adsorption effects. The synergy of multi-components in structural construction and interface interaction enables the material to achieve efficient removal of dyes and heavy metals.

To further systematically investigate the removal mechanism of alginate-based ternary composites for dyes, Table 4 comprehensively compares the adsorption capacity, removal efficiency (performance enhancement percentages were calculated using data obtained under identical experimental conditions within the same study), adsorption kinetics, and reusability of multiple materials. Additionally, the functional roles and synergistic mechanisms of the components involved in the removal process are specifically summarized.

Alginate-based ternary composites exhibit outstanding performance in dye wastewater treatment through multi-component synergy. Different components cooperate in structural construction, active-site provision, and interfacial regulation, enabling the material to possess a high specific surface area, porous structure, abundant surface functional groups, and a dense and stable three-dimensional network structure, thereby achieving a synergistic optimization of structure and function. In terms of the mechanism, such systems mainly manifest in two pathways. For systems dominated by adsorption, the efficient enrichment of dye molecules is mainly achieved through electrostatic interaction, hydrogen bonds, and π–π interactions. In the adsorption–catalysis synergy system, on the basis of adsorption enrichment, it further induces photocatalytic or reduction reactions through active components. The metal/metal oxide components can promote the capture and transport of photogenerated charge carriers through the surface plasmon resonance effect, accelerate the separation of electrons and holes, and improve the efficiency of light utilization efficiency, thereby significantly enhancing the degradation and transformation ability of pollutants. The ternary system demonstrates superior performance in terms of removal efficiency, reaction rate, and reusability, indicating great potential for practical applications.

### 3.3. Removal of Antibiotics

With the rapid development of the livestock farming and pharmaceutical industries, a large amount of antibiotics continuously enter the water environment through discharge or incomplete metabolism. Although their environmental concentration is usually only at the ng/L–μg/L level, antibiotics are characterized by high structural stability, resistance to degradation, and strong biological activity. The continuous existence of antibiotics will induce the generation and spread of antibiotic resistance genes (ARGs) and promote the formation of multidrug-resistant bacteria, thereby posing potential threats to ecosystem stability and human health [98]. In addition, antibiotics can accumulate in crops through agricultural irrigation, further increasing the risk of drug resistance. Based on the above environmental and health risks, the United States Environmental Protection Agency has included typical antibiotics such as sulfamethoxazole, tetracycline and erythromycin in the Pollutant Candidate List (CCL), emphasizing their potential risks to drinking water safety. Against this background, the development of new functional materials that can efficiently remove antibiotics is of great significance. Alginate-based ternary composites have shown promising application prospects in the field of antibiotic removal due to their advantages of multi-component synergy and adjustable structure.

Introducing inorganic non-metallic frameworks can enhance the adsorption and catalytic performance of composite systems. Yang et al. [99] synthesized Fe_3_O_4_@SiO_2_-NH_2_ nanoparticles using the reversed micelle-emulsion method and prepared the magnetic composite hydrogel Fe_3_O_4_@SiO_2_-NH_2_/SA by Fe^2+^ ionic crosslinking for the adsorption-coupled Fenton-like reaction (ACFLR) for the removal of tetracycline (TC) in water. The results showed that the adsorption capacity of TC reached as high as 588.41 mg/g at adsorption equilibrium (48 h), which was significantly higher than that of the single component (SA was 400 mg/g, Fe_3_O_4_@SiO_2_-NH_2_ was 50 mg/g), demonstrating a good synergistic enhancement effect. Under the coupled Fenton-like reaction conditions, the removal rate of TC within 4 h could reach 98.76%, further indicating that this system has significant advantages in the synergistic adsorption and catalysis. The system is mainly based on chemical adsorption, conforming to the pseudo-second-order kinetics and the Freundlich isothermal model. The removal process is driven by the multi-component synergy. SA forms a three-dimensional porous network structure, providing –COOH and –OH, and through electrostatic and hydrogen bonding interactions, adsorbs TC and fixes the magnetic components. Fe_3_O_4_@SiO_2_-NH_2_ further enhances the adsorption of TC through –NH_2_ groups, π–π interactions, and electrostatic interactions, and provides Fe^2+^/Fe^3+^ active centers, enabling H_2_O_2_ to diffuse in the porous network and react with Fe^2+^/Fe^3+^ to generate strong oxidative species such as ·OH, for the rapid degradation of the adsorbed TC. The system effectively improves the removal efficiency of antibiotics through the synergistic effect of adsorption and the Fenton-like reaction.

The combination of multiple natural polymers can optimize the structural stability and enhance the interfacial interaction of the composites. Their abundant functional groups and good gel-forming properties are conducive to the enrichment of pollutants and the promotion of subsequent reaction processes. Roy et al. [100] green-synthesized Fe_3_O_4_ and ZnO nanoparticles using green tea extract, and fixed them into the chitosan/sodium alginate (CS/SA) matrix through CaCl_2_ ionic crosslinking to prepare Fe_3_O_4_-ZnO-CS/SA nanocomposite microspheres. The material efficiently degraded ciprofloxacin (CIP) and sulfamethoxazole (SMX) in water under UV-C irradiation through adsorption–photocatalytic synergy. The experimental results showed that the optimal reaction conditions were pH 4.0 and an initial antibiotic concentration of 10 mg/L. When the microsphere dosage was 10 g for the CIP and 15 g for the SMX, the removal rates of CIP and SMX reached 94.78% and 93.32%, respectively. The system mainly relies on physical adsorption, which conforms to the pseudo-first-order kinetics and the Langmuir isothermal model. This indicates that antibiotics are mainly adsorbed as a uniform monolayer on the composite surface. CS/SA serves as the carrier, rich in –NH_2_, –OH and –COOH functional groups, and is adsorbed and enriched by electrostatic and hydrogen bonding interactions. ZnO acts as an excellent photocatalyst, generating electron–hole pairs upon light irradiation to drive redox reactions. Fe_3_O_4_ not only endows magnetic separability but also can inhibit the recombination of photogenerated charge carriers, improving the photocatalytic efficiency. Radical scavenging experiments further confirmed that singlet oxygen (^1^O_2_) and hydroxyl radical (•OH) are the dominant reactive oxygen species in the system. In addition, the composite has good reusability, demonstrating the comprehensive advantages of green synthesis, magnetic reusability, and the synergistic removal of antibiotic pollution through adsorption and photocatalysis.

Porous carbon-based materials possess a high specific surface area and π-conjugated structure, which can significantly enhance the enrichment and binding of organic pollutants. Prasannamedha et al. [101] used sugarcane bagasse as the raw material, and through hydrothermal carbonization and thermal activation obtained magnetic carbon (Fe/Fe_3_C/γ-Fe_2_O_3_). Then, they prepared magnetic hydrogel microspheres (SACFe) using sodium alginate as the matrix and CaCO_3_ as the crosslinking agent for removing sulfamethoxazole (SMX) from water. The results showed that under optimal conditions (pH = 6.2, dosage of 0.6 g/L), efficient removal of SMX at an initial concentration of 50 mg/L was achieved, with a maximum adsorption capacity of 58.439 mg/g. Kinetic and isothermal analyses indicated that this process conformed to the Elovich model and the Freundlich model, suggesting that the adsorption mainly occurred on a heterogeneous surface and was dominated by chemical adsorption. Furthermore, the Temkin model parameter (B > 0) indicates that the adsorption process is endothermic. Mechanism analysis indicated that the removal of SMX by SACFe was mainly driven by charge-assisted hydrogen bonding, π–π stacking interactions, and electron donor–acceptor (EDA) interactions. At pH = 6.2, SMX existed in an anionic form, while the –COOH groups underwent deprotonation to form –COO^−^, and the –OH groups partially remained protonated, thereby forming stable charge-assisted hydrogen bonds with the SMX molecules. Simultaneously, the aromatic ring of SMX could undergo π–π stacking with the conjugated structure of magnetic carbon, and the interaction is further strengthened by EDA effects. Moreover, in acidic or alkaline conditions, due to the same charge of SMX and the material surface, the electrostatic repulsion increased, thereby inhibiting the adsorption process. This also explains the phenomenon in which the removal efficiency gradually increased within the pH range of 3–6 and reached the optimal performance near neutral conditions.

From the perspective of component effects, each component played a significant synergistic role during adsorption. Fe/Fe_3_C/γ-Fe_2_O_3_ provided a high specific surface area and abundant π conjugated structure, which was the main contributor to π–π interactions and EDA interactions, and also endowed the material with magnetic separation properties. The SA matrix provided hydrogen bonding and electrostatic interactions through abundant –COOH and –OH groups and constructed a stable three-dimensional network to promote the diffusion and enrichment of pollutants. The synergistic effect of the components enhanced the adsorption capacity and interaction strength. However, after multiple cycles, the adsorption efficiency decreased (dropping to 61.4% in the fourth cycle), indicating that some active sites were irreversibly occupied or structurally damaged during the regeneration process.

To further systematically sort out the removal mechanism of alginate-based ternary composites toward antibiotics, Table 5 comprehensively compares the adsorption capacity, removal efficiency (performance enhancement percentages were calculated using data obtained under identical experimental conditions within the same study), adsorption kinetics and reusability of multiple materials. Additionally, the functional roles and synergistic mechanisms of the components involved in the removal process are specifically summarized.

Alginate-based ternary composites exhibit excellent removal performance in antibiotic removal. Natural polymer matrix mainly achieves the preliminary enrichment and interface regulation of pollutants by constructing a three-dimensional network structure and providing abundant functional groups. Porous carbon-based materials rely on high specific surface area, π-conjugated structure, or coordination sites to enhance intermolecular interactions, thereby improving the adsorption capacity. Metallic materials or metal oxide active centers further introduce electron transfer, the generation of reactive oxygen species, or Fenton-like/photocatalytic reaction pathways to achieve the transformation of adsorbed pollutants. In this synergistic system, the removal of antibiotics has evolved from the traditional single adsorption mode to a multi-pathway coupled mechanism, which significantly improves the efficiency of pollutant removal.

### 3.4. Removal of Other Pollutants

In addition to dyes, heavy metals and antibiotics, various complex pollutants are also widely present in the water environment, including microbial pollutants [102], pesticide residues, inorganic anions [103] and other emerging organic pollutants. These pollutants have the characteristics of diverse sources, complex structures and significantly different environmental behaviors, leading to their coexistence and interactions in water systems, which further increases the difficulty of treatment.

Among numerous complex pollutants, microbial contamination has significant biological hazards and diffusibility. Therefore, constructing a ternary composite system with antibacterial and controlled-release functions has become an important research direction. Vince Kovic et al. [70] prepared AgNPs + ZnONPs alginate microspheres by the ionic crosslinking method. SA serves as a three-dimensional matrix, providing hydrogen bonding and coordination sites through –COOH and –OH groups to regulate the swelling of microspheres and the dispersion of nanoparticles, achieving stable loading and controllable release. AgNPs loosen the microsphere structure and increase the swelling degree by weakening the hydrogen bonds in the alginate network, thereby achieving abnormal diffusion and release of Ag^+^. ZnONPs make the network denser and reduce swelling by enhancing the coordination of COO^−^–Zn^2+^, presenting Fick diffusion and zero-order controlled-release. Two-component systems can balance the structure and release behavior, generating a synergistic effect. The adsorption and antibacterial mechanisms mainly involve disruption of fungal cell membranes by nanoparticles, induction of reactive oxygen species (ROS) accumulation, and damage to cell wall polysaccharides. Under the synergistic effect, the antibacterial rate against Fusarium solani is as high as 99.7%, achieving a highly efficient, sustained-release, and safe antifungal effect.

Pesticides, as another type of organic pollutant, are widely present in water bodies due to their stable structure, strong toxicity and persistence in the environment, and are difficult to effectively remove by conventional water treatment processes. Therefore, the development of material systems with both adsorption and degradation functions is of great significance for the treatment of pesticide remediation. Liu et al. [64] adopted a green strategy without crosslinking agents to prepare Fe_3_O_4_@SiO_2_@Salg (GMS). The material exhibited removal efficiencies of 80.5–100% for 18 organophosphorus pesticides in tap water, river water, and seawater, with little interference from the water matrix and outstanding potential for practical application. After eight cycles, the removal efficiency remained above 80%. The adsorption conformed to the pseudo-second-order kinetics and Langmuir model, suggesting a dominant chemisorption mechanism. Fe_3_O_4_ endows magnetism to achieve rapid magnetic separation. SiO_2_ stabilizes the magnetic core, inhibits agglomeration and provides surface silanol groups. SA provides hydrogen bonding and electrostatic interactions through -COOH and -OH groups. The synergistic integration of these components enables efficient, stable, and safe adsorption of organophosphorus pesticides. Albqmi et al. [74] prepared SA/graphene oxide/CoMnFeO_4_ ternary composite hydrogel microspheres by the ionic crosslinking method to achieve efficient adsorption of dimethoate. The adsorption process conformed to the Freundlich isotherm and biexponential kinetic model, which was spontaneous, exothermic and multilayer physical adsorption. Under the optimal conditions, the removal rate reached 99.28% and remained above 80% after six cycles. A removal efficiency of 89.7% was achieved in real river water. SA serves as the gel matrix and provides hydrogen bonding and electrostatic interactions through -COOH and -OH groups. Graphene oxide provides a large specific surface area and π–π stacking with the aromatic rings of pesticides, enhancing the adsorption affinity. CoMnFeO_4_ provides magnetic separation ability and additional active sites, while improving the structural stability of the microspheres. The synergistic effect of these components results in high adsorption capacity, rapid kinetics, and facile separation for pesticide removal.

In summary, although the removal processes of different pollutants in alginate-based ternary composites exhibit certain differences, their adsorption mechanisms can all be attributed to the synergistic coupling of multiple interfacial interactions. The removal mechanisms include electrostatic interaction, coordination complexation, hydrogen bonding, ion exchange, porous physical enrichment, π-π interaction, redox reaction, and photocatalytic radical reaction. From the perspective of ternary composites, different components play clear functional roles and synergistic regulatory effects in the system. SA mainly provides a three-dimensional network structure and abundant –COOH and –OH functional groups, thereby achieving electrostatic interaction, coordination complexation, and hydrogen bonding, while improving mass transfer performance. Metal oxides, as functional components, can introduce specific active sites, endow photocatalytic, antibacterial, and magnetic separation properties, and provide additional active sites to enhance interfacial reactivity. The third component can be regulated according to the characteristics of the pollutants. Inorganic non-metallic frameworks are usually used as structural supports, construct stable porous skeletons, increase specific surface area and mechanical stability, regulate pore structure, and promote pollutant diffusion and enrichment. At the same time, they provide hydrogen bonding, complexation, electrostatic interaction, and π-π interaction sites through Si–OH and other oxygen-containing functional groups and effectively inhibit the agglomeration of nanoparticles. Natural polymers form stable three-dimensional crosslinked networks to provide structural support for the material and introduce –COOH, –OH, and –NH_2_ groups to enhance electrostatic interaction, coordination complexation, and hydrogen bonding, while improving mass transfer. Porous carbon-based materials, with their high specific surface area and π-conjugated structure, can significantly enhance the hydrophobic interaction, π–π stacking, and electron donor–acceptor interaction with organic pollutants, thereby strengthening adsorption capacity and interfacial binding strength. Metal materials provide catalytically active centers or electron transfer channels, and can further oxidize and degrade adsorbed pollutants through Fenton-like reactions, photocatalytic processes, or radical generation mechanisms. The advantages of the ternary composite system are not only reflected in the improvement of pollutant removal efficiency, but more importantly, they achieve multi-mechanism synergistic regulation capabilities through structural designability, enabling the material to achieve differentiated and efficient removal of pollutants with different properties.

### 3.5. Comparative Evaluation of Binary and Ternary Alginate-Based Composites

Numerous studies have demonstrated that ternary composites typically exhibit superior pollutant removal performance compared with binary composites. Table 6 further compares the performance changes in alginate-based binary and ternary composites (performance enhancement percentages were calculated using data obtained under identical experimental conditions within the same study). However, a systematic analysis of the existing literature reveals several limitations in the existing comparisons. Most studies mainly focus on the performance optimization of the final ternary composites, but lack systematic comparisons with the corresponding binary composites under the identical experimental conditions. Consequently, the actual contribution of the third component to adsorption capacity, specific surface area, and mechanical properties cannot be accurately quantified. Currently reported performance advantages more reflect the optimization results of individual material systems under specific experimental conditions rather than the absolute contribution of the third component itself. Future research should further establish a standardized control system and increase binary control experiments to more objectively evaluate the real advantages of different ternary composite strategies.

### 3.6. Synergistic Mechanisms of Alginate-Based Ternary Composites

Although the enhanced performance of alginate-based ternary composites has been attributed to the “synergistic effect”, the explanations of the synergistic mechanism in different studies are rather scattered and lack a unified mechanistic framework. Based on the research results reviewed in this section, the synergistic mechanisms can be summarized into three categories: structural synergy, interfacial synergy, and functional synergy.

Firstly, structural synergy is primarily reflected in the regulatory effect of the third component on the composite’s microstructure. Inorganic non-metallic frameworks, metal nanoparticles, natural polymer materials, and carbon-based materials can all compensate for the structural limitations of alginate/metal oxide binary systems. By increasing the specific surface area, constructing a multi-level pore structure promotes the uniform dispersion of metal oxides, and inhibits particle agglomeration, exposing more active sites and improving the diffusion and mass transfer processes of pollutants within the composite. Meanwhile, the stable three-dimensional network structure can effectively reduce the leaching of metal oxides, and enhance the mechanical stability and reusability of the composite. Therefore, this type of synergy is mainly manifested in enhancing the utilization rate of active sites and structural stability, without altering the intrinsic removal mechanisms of pollutants.

Secondly, interfacial synergy stems from the formation of stable interfacial interactions among the three components. Sodium alginate provides abundant -COOH and -OH. The hydroxyl groups on the surface of metal oxides can offer coordination-active sites, while the third component further introduces new active sites such as silanol groups, amino groups, oxygen-containing functional groups, or aromatic structures. These functionalities enhance the binding between components through hydrogen bonding, electrostatic interactions, coordination, and interfacial electron interactions. Thus, a more stable interface structure is formed. During the adsorption process, multiple adsorption mechanisms often operate cooperatively, including electrostatic attraction, surface complexation, ion exchange, hydrogen bonding, and π–π interactions, enabling adsorption capacity and improved selectivity of the composite.

Finally, functional synergy is a key feature that distinguishes ternary composites from binary systems. The third components also endow the materials with new functions. For instance, Fe_3_O_4_ offers rapid magnetic separation capabilities, semiconductors such as TiO_2_ and ZnO provide photocatalytic degradation capabilities, metal nanoparticles can promote electron transfer and catalytic reduction, while natural polymers further enhance the mechanical properties and stability of the composite. Therefore, pollutants are first enriched on the material surface through adsorption, and then further undergo photocatalytic oxidation, catalytic reduction or magnetic separation for recovery, achieving multi-functional synergistic removal and enhancing the overall treatment efficiency and material recycling performance.

A further comparison of the removal mechanisms of different pollutants reveals that the contributions of multiple mechanisms vary considerably. For heavy metal ions, surface complexation and ion exchange dominate the removal process, while electrostatic adsorption is mainly affected by the pH of the solution and the surface charge of the material, playing an auxiliary promoting role. For organic pollutants such as dyes, electrostatic adsorption is usually dominant, accompanied by hydrogen bonding and π–π interactions. The optimization of the specific surface area and pore structure of the third component further promotes the diffusion of pollutants and the utilization of active sites. For photocatalytic systems, adsorption is mainly responsible for the enrichment of pollutants, while photocatalytic oxidation becomes the main mechanism for subsequent deep degradation. The coupling of these two processes enables a continuous synergistic removal mechanism, thereby improving the overall removal efficiency.

It is worth noting that the “synergy effect” frequently mentioned in the literature does not necessarily belong to true synergy. In some systems, the third component merely increases the specific surface area or provides additional adsorption sites, and the resulting performance enhancement is essentially an additive effect. True synergistic effects can only be considered to occur when the incorporation of the third component alters the interfacial properties of the composite, introduces new removal mechanisms, or enables the coupling of multiple functions, thereby allowing the overall performance of the ternary system to exceed the sum of the individual contributions of its components.

### 3.7. Critical Evaluation of Alginate-Based Ternary Composite Strategies

Although significant progress has been made in sodium alginate-based ternary composites, existing research mainly focuses on improving the performance of individual composite systems, lacking systematic comparisons among different ternary composite strategies. Therefore, the superior performance reported in the literature cannot be simply used to evaluate the superiority or inferiority of a certain strategy, but should be comprehensively analyzed in combination with the material composition, target pollutants, and experimental conditions. Due to the significant differences in the types of pollutants, initial concentrations, solution pH, adsorbent dosage and testing methods adopted by different research, there is a lack of unified comparability in adsorption capacity and removal efficiency. Furthermore, many studies attribute the performance improvement to the “synergistic effect”, but such conclusions are largely based on qualitative improvements in adsorption performance, lacking quantitative analysis of the contributions of each component and the synergistic mechanism. Therefore, the enhanced performance observed in some ternary systems may be more appropriately interpreted as an additive effect rather than a true synergistic effect.

Based on the available research, the functional roles of different third components in the ternary system are significantly different. Therefore, their main advantages and limitations are summarized in Table 7. Inorganic non-metallic frameworks are more focused on improving structural stability and pore structure. Carbon-based materials can increase the specific surface area and the adsorption capacity of organic pollutants, but the preparation cost and agglomeration issues still need to be addressed. Metal nanoparticles have multi-functional properties, such as magnetic separation and photocatalysis, but their long-term stability and the risk of metal leaching still deserve attention. Natural polymers are mainly used to improve interfacial compatibility and mechanical properties, but their contribution to adsorption activity is relatively limited. Therefore, there is currently no optimal ternary composite strategy applicable to all pollutants. Future material design should involve the rational selection of the third component according to the characteristics of the target pollutants, together with the establishment of a more standardized performance evaluation framework to enable more objective and reliable comparisons among different composite systems.

In addition, when comparing different alginate-based ternary systems, it is also necessary to pay attention to some common issues existing in the current literature. Although different sodium alginate-based ternary composite systems all demonstrate outstanding performance in removing heavy metals, dyes and other pollutants, it remains difficult to determine whether any particular strategy offers a universal advantage. Firstly, there are significant differences in the types of the third component, target pollutants, experimental conditions (e.g., initial concentration, solution pH, adsorbent dosage and reaction time), as well as performance evaluation indicators adopted by different studies. As a result, the reported adsorption capacities and removal efficiencies are often not directly comparable. Therefore, the performance differences between different systems cannot be simply attributed to the differences in material composition. Secondly, performance enhancement is frequently ascribed to “synergistic effects”; most studies only provide qualitative interpretations based on experimental observations, lacking quantitative analysis of the contributions of each component and the synergistic mechanism. Therefore, the reported synergistic effects in some studies may be more appropriately interpreted as structural optimization or functional integration, rather than a true synergistic enhancement.

Furthermore, most studies are still mainly based on single-pollutant systems under laboratory conditions, and the evaluation of complex actual wastewater, multi-component pollutant coexistence environments, and the long-term reusability of materials is relatively limited. Regeneration and reusability are important indicators for evaluating the practical application potential of alginate-based ternary composites. Common regeneration methods include acid or alkali washing, salt solution-induced desorption, organic solvent extraction, and photocatalytic regeneration. Acidic solutions usually promote the desorption of metal ions by protonating binding sites such as carboxyl and hydroxyl groups. Salt solutions mainly achieve the desorption of pollutants through ion exchange and competitive binding. Alkaline solutions may promote the release of certain anionic pollutants, while organic solvents are commonly used to desorb dyes and other organic contaminants. For photocatalytically active composites, the adsorbed organic pollutants can be degraded in situ under irradiation, thereby restoring the active sites and reducing the generation of high-concentration desorption effluents. However, the suitability of different regeneration methods depends on the type of pollutant, the binding mechanism between the pollutant and the composite, and the chemical stability of the composite. There are significant differences in the regeneration performance reported by existing research, mainly due to the regenerant type and concentration, desorption time, number of cycles, and reusability evaluation criteria. Many studies only report the removal efficiency after a limited number of cycles, making it difficult to directly compare the results of different studies. Therefore, standardized indicators, including desorption efficiency, adsorption capacity retention, number of cycles, adsorbent mass loss, and regenerant consumption, should be used to evaluate regeneration performance.

Moreover, repeated adsorption–desorption cycles may alter the composite structure. Acidic, alkaline or high-ionic-strength regenerants may cause swelling or shrinkage of the alginate network, cross-linked ions to be displaced or lost, weakening of the egg-box structure, pore collapse, components to leach out, and the mechanical integrity to decline. The irreversible occupation of active sites by pollutants may also lead to a gradual decline in adsorption performance. However, most studies only evaluate reusability solely based on changes in removal efficiency or adsorption capacity, and have not systematically investigated the structural stability before and after cycling. Therefore, performance changes observed after a limited number of cycles remain insufficient to comprehensively assess the long-term regeneration capacity and practical applicability of these materials.

## 4. Limitations and Future Research Recommendations

Although the alginate-based ternary composites exhibit significant advantages in terms of structural designability, functional synergy, and pollutant removal efficiency, there are still notable limitations in practical applications. Firstly, from the perspective of material properties, the composites usually require multi-step synthesis, such as ionic crosslinking, in situ loading, and carbonization. Such procedures are complex and energy-intensive, which increases costs and limits their large-scale application. Additionally, the system involves different types of materials such as organic matrices, inorganic components, and carbon-based materials. The interfacial compatibility between different components is insufficient, and the interfacial bonding strength is limited, which can easily lead to weak interface bonding and potential problems such as delamination or cracking, thereby affecting the effective utilization of adsorption sites and the overall performance stability. Moreover, certain inorganic components may undergo slight leaching or surface structure degradation in aqueous environments, resulting in a gradual decline in material performance during reuse and even potential secondary pollution risks. Beyond these technical challenges, the economic feasibility of alginate-based ternary composites also deserves careful consideration. In general, sodium alginate and Ca^2+^-mediated ionic crosslinking are inexpensive, whereas the third component is the primary factor influencing the overall material cost. Natural and low-cost materials, such as bentonite, biochar, and silica, offer clear economic advantages, whereas high-performance materials, including graphene derivatives, MXenes, and noble metal nanoparticles, can substantially enhance material performance but also significantly increase production costs. Moreover, additional processing steps, such as freeze-drying and complex surface modification, further increase energy consumption and fabrication costs. Therefore, future research should place greater emphasis on achieving an optimal balance between material performance, economic feasibility, and scalability by preferentially employing low-cost third components and simple, environmentally friendly, and scalable fabrication strategies.

Secondly, in terms of application and technology, there is still a significant gap between the excellent performance obtained under laboratory conditions and the actual effectiveness in complex real water bodies. In actual water bodies, the adsorption process is not only affected by factors such as the initial pollutant concentration, solution pH and temperature, but also easily interfered with by complex components such as high salinity, coexisting ions and natural organic matter, thereby weakening the multi-mechanism synergy and reducing the selectivity and competitive adsorption capacity. The performance of composites may also significantly decline. In addition, alginate-based ternary composites demonstrate considerable competitive potential compared with conventional commercial adsorbents (such as activated carbon, zeolites, and ion exchange resins), particularly in terms of adsorption capacity, tunable surface functionality, reusability, and multi-functional integration. However, the available evidence remains insufficient to establish that their overall performance is superior to that of mature industrial adsorbents. Conventional commercial adsorbents benefit from standardized production processes, established scalability, high mechanical durability, and well-developed continuous-flow applications. In contrast, most sodium alginate-based ternary composites remain at the laboratory research stage. Their long-term mechanical stability, swelling behavior, risk of functional component leaching, long-term regeneration performance, treatment efficiency in real wastewater, and large-scale production costs have yet to be systematically evaluated.

Based on the above limitations, future research on alginate-based ternary composites can be improved and expanded in multiple aspects. In terms of material design, the bonding strength of the three-phase interface can be enhanced through interface modification and crosslinking reinforcement strategies, and a multi-level pore structure can be constructed by combining pore structure regulation. At the same time, multiple functional groups can be introduced to improve the selectivity and anti-interference ability of the material. In terms of preparation processes, interface engineering and precise assembly strategies should be developed to optimize component distribution and enhance structural stability. At the same time, low-energy consumption and simplified one-step technology should be promoted. Functional components derived from agricultural and forestry wastes can be utilized to reduce costs, and continuous production processes should be further explored to enable large-scale preparation. In terms of applied and theoretical research, particular attention should be paid to the adaptability of materials under real water conditions, including their performance in complex ionic environments, organic–inorganic coexistence systems, and continuous-flow dynamic systems, in order to promote their transformation from laboratory research to engineering applications. At the same time, the full life cycle assessment and solid waste resource utilization strategies should be combined to enhance the environmental friendliness and sustainable application potential.

Finally, the resource utilization of waste materials after reuse also deserves special attention. A recycling and regeneration system for adsorbents should be established, and their secondary applications in fields such as antibacterial, catalytic or energy conversion should be explored to achieve high-value utilization of materials and minimize waste. Therefore, developing low-cost, scalable and environmentally friendly ternary composite systems and enhancing their stability and functional response capabilities in complex environments will be the key development direction for their engineering application in water pollution treatment.

## 5. Summary and Outlook

The review focuses on alginate-based ternary composites as the research object, systematically summarizes their synthesis strategies and structural characteristics, and deeply explores their synergistic mechanism and performance advantages in pollutant removal. Compared with the alginate-based binary composites, ternary composites have achieved multi-mechanism coordinated regulation by introducing a third component, which is markedly improved in terms of structural stability, specific surface area, and pore structure regulation.

From the perspective of material construction, alginate, as a natural polymer matrix, builds a stable three-dimensional network framework relying on its “egg box structure” and provides abundant carboxyl and hydroxyl functional groups, serving as the foundation for pollutant enrichment and interfacial reactions. Metal oxides, as the core functional units, can endow composites with multiple properties such as adsorption, photocatalysis, oxidation-reduction and magnetic separation. The third component (such as inorganic non-metallic frameworks, metallic materials, porous carbon-based materials, and natural polymers) plays a key role in structural regulation, interface enhancement, mass transfer optimization and functional expansion.

For the reaction mechanism, pollutant removal by alginate-based ternary composites no longer relies on a single process, but instead reflects the synergistic coupling of multiple mechanisms, including electrostatic adsorption, coordination complexation, ion exchange, hydrogen bonding, porous structure enrichment, and π–π interaction. Meanwhile, photocatalysis, antibacterial or REDOX reactions are introduced into specific systems, realizing the further degradation and transformation of pollutants. This shift from “adsorption-dominated” to “catalytic or reduction synergy” has significantly enhanced the adaptability of the materials to complex pollutant systems.

From an application perspective, alginate-based ternary composites have demonstrated outstanding performance in the removal of various typical pollutants such as heavy metals, organic dyes and antibiotics. For different types of pollutants, differentiated responses and efficient removal can be achieved by regulating the composition and structural characteristics of materials. Overall, the ternary system outperforms the traditional system in terms of removal efficiency, reaction rate and reusability, demonstrating a promising engineering application prospect.

In summary, alginate-based ternary composites can efficiently remove various types of pollutants in water bodies through structural regulation and multi-mechanism synergy. Their development is evolving from simple composite construction to fine structural regulation and functional integration. Future research should focus on strengthening interfacial interactions, refining and scaling up preparation processes, and further evaluating the adaptability of materials under real water conditions, including complex ionic environments, organic–inorganic coexistence systems, and continuous-flow dynamic systems, to promote their effective transformation from laboratory research to engineering applications.

## Figures and Tables

**Table 1:** Comparison of commonly used ionic crosslinkers for sodium alginate.

Crosslinker	Concentration	Crosslinking Time	Characteristics	Influence on Properties
CaCl_2_	1–5 wt%	10–60 min	Most widely used; mild ionic crosslinking	Good biocompatibility, balanced mechanical strength and porosity
BaCl_2_	1–5 wt%	10–60 min	Stronger affinity to G blocks than Ca^2+^	Higher mechanical strength and stability, but higher toxicity
ZnCl_2_	0.5–2 wt%	10–60 min	Functional divalent crosslinker	Improved adsorption activity and antibacterial properties; excessive Zn^2+^ may reduce biocompatibility
FeCl_3_	0.1–1 wt%	5–30 min	Trivalent ion with strong coordination ability	Higher crosslinking density and catalytic functionality, but may produce brittle gels and color changes
AlCl_3_	0.1–1 wt%	5–30 min	High-valence ionic crosslinker	Increased gel rigidity and structural stability; excessive crosslinking may reduce swelling and diffusion

**Table 2:** Comparison of different construction strategies for alginate-based ternary composites.

Construction Strategy	Advantages	Limitations
Physical mixing + ionic crosslinking	Simple preparation, low cost, scalable	Weak interfacial interaction, possible aggregation
Self-assembly + ionic crosslinking	Strong interfacial integration, uniform distribution	More complex synthesis conditions
Co-precipitation + ionic crosslinking	Good dispersion of nanoparticles, enhanced structural integration	Particle size and crystallinity are sensitive to reaction conditions
In situ loading + ionic crosslinking	High loading efficiency, improved active-site utilization	Multi-step preparation, relatively complicated process

**Table 3:** Performance comparison of alginate-based ternary composites for heavy metals.

Pollutant	Composites	Experimental Conditions	Adsorption Capacity Removal Efficiency	Performance Enhancement	Reusability	Kinetics Isotherm Models	Main Mechanism	Functional Role of Components	Ref
Cd^2+^	SA/Fe_3_O_4_/ Bio-SiO_2_	pH 7.0 25 °C C_o_ = 10–320 mg/L dosage = 1.0 g/L	q_max_ = 35.36 mg/g 79.23%	—	52.6% after 5 cycles	PSO, Langmuir (monolayer chemisorption, spontaneous exothermic)	Coordination, electrostatic interaction, physical enrichment, ion exchange	SA: 3D network with –COOH/–OH for coordination and ion exchange. Bio-SiO_2_: Porous structure increases SSA; prevents NP aggregation. Fe_3_O_4_: Magnetic separation and active sites.	[78]
Cu^2+^	SA/ZnO/ NH_2_-Fe (III)	pH 4.0 25 °C C_o_ = 50–1500 mg/L dosage = 1.0 g/L	2144.5 mg/g 98.25%	SA/ZnO/Fe(III): 1266 mg/g Increase 70%	—	PSO, Langmuir (monolayer chemisorption, spontaneous exothermic)	Electrostatic interaction, coordination, surface deposition, H-bonding	SA: 3D network with –COOH/–OH for coordination and ion exchange. Fe(III)/ZnO: Provides abundant active sites. NH_2_-Fe(III): Enhances electrostatic and coordination binding.	[79]
Tl^+^	SA/MnO_2_ + Fe_3_O_4_ /CMC	pH 6.0 30 °C C_o_ = 0.7–214 mg/L dosage = 4.0 g/L	38.8 mg/g 100%	MnO_2_:20.1 mg/g Increase 93%	83.9% after 5 cycles	PSO, Freundlich (multilayer chemisorption, spontaneous endothermic)	Redox, ion exchange	SA/CMC: Porous matrix for MnO_2_ immobilization. MnO_2_: Redox mediator. Fe_3_O_4_: Magnetic separation and ion exchange.	[72]
Ag(I), Co(II) and Ni(II)	Cs@CA-CMC/ Fe_2_O_3_-CuO	pH 7.0 25 °C C_o_ = 5 mg/L dosage = 1.0 g/L	Ag(I): 4.91 mg/g Co(II): 4.5 mg/g Ni(II): 3.0 mg/g	—	70% 3 cycles	PSO, Langmuir (monolayer chemisorption, spontaneous exothermic)	Coordination bonding, electrostatic interaction, ion exchange	CS: Chelation of metal ions via –NH_2_/–OH groups. CMC: Ion exchange through –COO^−^ groups. CA: Porous support matrix for adsorption. Fe_2_O_3_-CuO: Magnetic recovery and additional active adsorption sites.	[81]
Pb^2+^	CS/SA/Fe_3_O_4_ @SiO_2_	pH 4.2 20 °C C_o_ = 20–500 mg/L dosage = 0.83 g/L	234.77 mg/g 99.04%	—	82% 3 cycles	Elovich, Langmuir (monolayer chemisorption)		CS: Chelation of metal ions via –NH_2_/–OH groups. SA: 3D network with –COOH/–OH for coordination. Fe_3_O_4_: Magnetic separation. SiO_2_: Provide stability and additional active sites.	[47]
Cr^6+^	SA/Fe_3_O_4_/ BC	pH 2.0 25 °C C_o_ = 10–350 mg/L dosage = 1.0 g/L	316.25 mg/g	—	92.5% after 7 cycles	PSO, Langmuir (monolayer chemisorption, spontaneous endothermic)	Electrostatic interaction, redox, coordination	SA: 3D network with –COOH/–OH for coordination and ion exchange. BC: High SSA/porosity; drives redox (Cr(VI)→Cr(III)). Fe_3_O_4_: Magnetic separation and active sites.	[85]
Pb^2+^	SA/Fe_3_O_4_/ GO	pH 6.0 28 °C C_o_ = 20–50 mg/L dosage = 0.25 g/L	270.27 mg/g 97.82%	—	82.28% after 5 cycles	PSO, Langmuir Freundlich (monolayer and multilayer adsorption, chemisorption)	Electrostatic interaction, coordination, ion exchange	SA: 3D network with –COOH/–OH for coordination and ion exchange. GO: High SSA/functional groups for binding. Fe_3_O_4_: Magnetic and active sites.	[84]
Sb(V)	Fe/(MgFe_2_O_4_-BC) /SA	pH 5 ± 0.5 25 ± 1 °C C_o_ = 10–300 mg/L dosage = 1.0 g/L	125.65 mg/g	twice that of Fe/MgFeO	—	Elovich, Freundlich (chemisorption and non-homogeneous)	Electrostatic interaction, ligand exchange, inner-sphere complexation and hydrogen bonding	SA: 3D network with –COOH/–OH for electrostatic interaction. BC: Dispersion and support. MgFe_2_O_4_: -OH groups for ligand exchange, hydrogen bonding, inner-sphere complexation and provides magnetic separation performance.	[43]

**Table 4:** Performance comparison of alginate-based ternary composites for dyes.

Pollutant	Composites	Experimental Conditions	Adsorption Capacity Removal Efficiency	Performance Enhancement	Reusability	Kinetics Isotherm Models	Main Mechanism	Functional Role of Components	Ref
MB	SA/TiO_2_/ Bnt	room temperature nature pH C_o_ = 320 mg/L dosage = 2.8 g/L	112 mg/g 98%	SA: 83% Increase 16%	TiO_2_ leaching < 2 ppm after 5 cycles	—	Electrostatic interaction, porous enrichment, ion exchange, photocatalysis	SA: 3D network with –COOH/–OH for coordination and electrostatic interaction. Bnt: Structural enhancement and enrichment. TiO_2_: Photocatalytic mineralization (e^−^–h^+^/·OH).	[68]
MB	SA@Fe_3_O_4_@MCM-41	room temperature nature pH C_o_ = 6 mmol/L catalyst mass = 4.8 mg V = 3.5 mL NaBH_4_ = 0.6 mol/L	5 min 100%	—	5 cycles	—	Electrostatic interaction Electron transfer Catalytic reduction Pore diffusion	NaBH_4_: Electron donor/reducing agent. Fe_3_O_4_: Catalytic active center. MCM-41: Mesoporous dispersion support. SA: 3D network with –COOH/–OH for electrostatic interaction.	[63]
Basic blue11 Acid red 138	SA/ZnO/ Ag	room temperature nature pH C_o_ = 100 mg/L catalyst mass = 1 g V = 40 mL	Basic blue11: 3.8 mg/g 95% Acid red 138: 3.4 mg/g 85%	—	Basic blue11: 80% Acid red 138: 75% after 4 cycles	PFO, Langmuir (Monolayer physisorption)	Electrostatic interaction, coordination, catalytic reduction	SA: 3D network with –COOH/–OH for coordination and electrostatic interaction. ZnO: Semiconductor catalyst. Ag: Facilitates electron transfer.	[96]
MB	SA/Zr/TiO_2_	pH 6.0 room temperature Co = 5–20 mg/L dosage = 25.0 g/L	40.48 mg/g	—	Decreased by 24% after 3 cycles	PSO (Exothermic spontaneous chemisorption)	Electrostatic interaction, H-bonding	SA: 3D network with –COOH/–OH for electrostatic interaction, H-bonding. Zr/TiO_2_: Increase the specific surface area and active sites.	[89]
MB	SA/Fe_3_O_4_/ CS	pH 10.0 25 °C C_o_ = 25–150 mg/L dosage = 0.3 g/L	526.32 mg/g 75.03%	SA: 276.2 mg/g Increase 90%	71% after 3 cycles	PSO, Langmuir (Monolayer chemisorption)	Electrostatic interaction, H-bonding, pore adsorption	CS/SA: –NH_2_/–OH/ -COOH groups for H-bonding and electrostatic interaction. Fe_3_O_4_: Magnetic separation and active sites.	[92]
MO	CuO-Fe_2_O_3_@SA-CMC-CS	room temperature nature pH C_o_ = 0.01 Mm catalyst mass = 5–6 mg NaBH_4_ = 0.2 mol/L	94% 3 min	—	3 cycles	PFO physisorption	Electrostatic interaction, H-bonding, electron transfer	SA-CMC-CS:3D network with –NH_2_/–COOH/–OH/for electrostatic interaction, H-bonding. CuO-Fe_2_O_3_: The core of the catalytic reaction and electron transfer.	[91]
MB	SA/Fe_3_O_4_/ BC	pH 6.0 room temperature C_o_ = 50–150 mg/L dosage = 2.0 g/LC_0_ = 100	153.2 mg/g 98%	—	—	PFO, Freundlich (Multilayer physisorption)	Electrostatic, hydrophobic, H-bonding, π–π stacking	SA: –OH/-COOH groups for H-bonding and electrostatic interaction. BC: High SSA and Porous structure; provides π-electron system. Fe_3_O_4_: Magnetic separation and active sites.	[97]

**Table 5:** Performance comparison of alginate-based ternary composites for antibiotics.

Pollutant	Composites	Experimental Conditions	Adsorption Capacity Removal Efficiency	Performance Enhancement	Reusability	Kinetics Isotherm Models	Main Mechanism	Functional Role of Components	Ref
TC	SA/Fe_3_O_4_/ SiO_2_-NH_2_	pH 4.0 25 °C C_o_ = 60 mg/L dosage = 20.2 g/L H_2_O_2_ = 100 μL	588.41 mg/g 98.76%	SA: 400 mg/g Increase 47%	—	PSO, Freundlich (multilayer chemisorption)	π–π stacking, H-bonding, electrostatic, Fenton-like	SA: –OH/–COOH groups for H-bonding and electrostatic interaction. Fe_3_O_4_@SiO_2_-NH_2_: π–π/electrostatic binding; Fe^2+^/Fe^3+^ Fenton-like active centers.	[99]
TC	AC/FeMnMCM-41/SA	pH 5.44 30 °C C_o_ = 10 mg/L dosage = 0.66 g/L H_2_O_2_ = 5%	90 min 91%	The removal rate is 4.3 times that of SA/FeMnMCM-41	33 cycles	PFO physisorption	π–π interaction: electron transfer, redox cycling ROS generation H-bonding electrostatic interaction	AC: π–π interaction and pore adsorption. FeMnMCM-41: Electron transfer, redox cycling and ROS generation. SA:–OH/–COOH groups for H-bonding and electrostatic interaction.	[98]
CIP SMX	SA/Fe_3_O_4_-ZnO/ CS	pH 4 room temperature C_o_ = 10 mg/L dosage = 10 g (CIP) and 15 g (SMX) H_2_O_2_ = 5%	CIP: 94.77% SMX: 93.31%	—	CIP: 41.72% SMX: 46.02% after 5 cycles	PFO, Langmuir (monolayer physisorption)	Photocatalysis electrostatic, H-bonding	SA/CS: –NH_2_/–OH/ -COOH groups for H-bonding and electrostatic interaction. .ZnO: Photocatalyst. Fe_3_O_4_: Magnetic separation; inhibits carrier recombination.	[100]
SMX	SA/Fe/Fe_3_C/γ-Fe_2_O_3_/BC	pH 6.2 room temperature C_o_ = 50 mg/L dosage = 0.6 g/L	58.44 mg/g 98.32%	—	61.4% after 4 cycles	Elovich Freundlich Temkin (B > 0) (heterogeneous surface endothermic chemisorption)	H-bonding, electrostatic, EDA, π–π stacking	SA: –OH/–COOH groups for H-bonding and electrostatic interaction. Fe/Fe_3_C/γ-Fe_2_O_3_: High SSA and π-conjugation for EDA/π–π interactions; magnetic separation.	[101]

**Table 6:** Quantitative comparison of binary and ternary alginate-based composites for water treatment.

Pollutant	Ternary Composite and the Third Component	BET Surface Area (m^2^/g)	Experimental Conditions	Adsorption Capacity (mg/g) or Removal Efficiency (%)	Mechanical Strength	Ref
Cd^2+^	SA/Fe_3_O_4_/Bio-SiO_2_	/	/	/	The ternary composite formed a more integrated three-dimensional network structure, strengthened the interfacial interactions among the components, and improved the structural stability of the composite.	[78]
Cu^2+^	SA/ZnO/NH_2_-Fe (III)	/	pH 4.0 25 °C C_o_ = 50–1500 mg/L dosage = 1.0 g/L	1266 to 2144.5 mg/g (69.4% increase)	SiO_2_ provided a stable support, while amine functionalization introduced additional active sites, jointly improving the structural integrity and adsorption activity.	[79]
Ti^+^	SA/MnO_2_ + Fe_3_O_4_/CMC	/	/	/	CMC improved MnO_2_/Fe_3_O_4_ dispersion and promoted an open porous network.	[72]
Cr^6+^	SA/Fe_3_O_4_/BC	/	/	/	Biochar provided a porous framework, while alginate stabilized Fe_3_O_4_ nanoparticles, improving structural stability and reusability.	[85]
MB	SA/TiO_2_/Bnt	/	room temperature nature pH C_o_ = 320 mg/L dosage = 2.8 g/L	88%to 96% (8% increase)	Bentonite provided a porous framework, promoted uniform TiO_2_ dispersion, and enhanced structural stability and adsorption performance.	[68]
Basic blue11 Acid red 138	SA/ZnO/Ag	/	/	/	Ag was uniformly incorporated into the ZnO/alginate network, enhancing interfacial interaction and electron transfer to form a stable catalytic composite.	[96]
MB	SA/Fe_3_O_4_/CS	/	/	/	Chitosan enhanced porosity and mechanical stability, alginate provided a three-dimensional crosslinked network with abundant carboxyl groups, and Fe_3_O_4_ imparted magnetic separability, collectively forming a stable, efficient, and recyclable adsorption system.	[92]
MB	SA/Fe_3_O_4_/BC	/	/	/	Activated biochar provided a high specific surface area, abundant mesopores, and additional active adsorption sites, thereby enhancing mass transfer and adsorption performance.	[97]
TC	SA/Fe_3_O_4_/SiO_2_-NH_2_	21.15 to 54.98 m^2^/g (160% increase)	pH 4.0 25 °C C_o_ = 60 mg/L dosage = 20.2 g/L H_2_O_2_ = 100 μL	30 to 588.41 mg/g (36.8% increase)	SiO_2_ increased the specific surface area and pore volume, while amino groups introduced additional active sites, promoting uniform Fe_3_O_4_ dispersion and enhancing the structural stability and adsorption activity of the composite.	[99]
CIP SMX	SA/Fe_3_O_4_-ZnO/CS	/	/	/	Chitosan introduced abundant amino groups, strengthened the interfacial interaction with alginate, and provided additional active sites for antibiotic adsorption.	[100]
SMX	SA/Fe/Fe_3_C/γ-Fe_2_O_3_/BC		/		Magnetic biochar acted as a rigid framework, improving the mechanical stability of alginate hydrogels, suppressing swelling, and providing additional active adsorption sites.	[101]

**Table 7:** Critical comparison of different alginate-based ternary composite strategies for water treatment.

Ternary Strategy	Main Contribution	Advantages	Limitations
Inorganic non-metallic framework	Improve pore structure and structural stability	Good mechanical strength, enhanced stability, and suppressed nanoparticle aggregation	Limited intrinsic adsorption activity; performance mainly relies on synergistic components
Carbon-based materials	Increase specific surface area and introduce π-conjugated structures	High adsorption capacity, enhanced electron transfer, and favorable for organic pollutants	High cost, aggregation/restacking, preparation complexity
Metal nanoparticles	Provide magnetic separation, photocatalysis or catalytic reduction	Multi-functionality and high removal efficiency	Possible nanoparticle aggregation, metal leaching, and long-term stability require further evaluation
Natural polymer materials	Improve compatibility, flexibility and structural integrity	Good biocompatibility, enhanced mechanical properties	Limited direct contribution to adsorption; usually requires a combination with other functional components

## Data Availability

The original contributions presented in this study are included in the article. Further inquiries can be directed to the corresponding authors.
